# Hepatitis B virus promotes hepatocellular carcinogenesis by activating IL-6-dependent tumor-macrophage crosstalk and M2-like macrophage polarization

**DOI:** 10.1016/j.jbc.2026.113283

**Published:** 2026-06-26

**Authors:** Yingqi Liu, Xinyi Cao, Bingbing Yang, Hongmei Zheng, Hongyu Zhong, Dan Tao, Menghuan Li, Zhong Luo

**Affiliations:** 1 School of Life Science, Chongqing University, Chongqing, P. R. China; 2 Department of Radiation Oncology, Chongqing University Cancer Hospital, Chongqing, P. R. China

**Keywords:** antitumor immune responses, HBx, hepatocellular carcinoma, M2-like macrophages, tumor immune microenvironment, AIM2 inflammasome, IL-6 signaling

## Abstract

Hepatitis B virus X protein (HBx) is a prominent promoter of hepatitis B virus (HBV)-induced hepatocellular carcinoma (HCC) in the clinic, but the associated pro-tumorigenic mechanisms are still not well elucidated, especially its potential involvement in the tumor immunoevasion and immunotolerance. Surprisingly, we found that HBx expression status of HCC cells showed marked positive correlation with its interleukin 6 (IL-6) secretion, which drove M2 macrophage polarization *via* activating signal transducer and activator of transcription 3 (STAT3) signaling and fostered an immunosuppressive tumor microenvironment (TME). During this process, HBx partially translocated into mitochondria to cause mitochondrial DNA (mtDNA) leakage and activate absent in melanoma 2 (AIM2)-IL1β-nuclear factor kappa-B (NF-κB) signaling for boosting tumor-intrinsic IL-6 production. In line with these mechanistic insights, blocking IL-6 signaling in HBx-positive HCC tumors significantly revitalized the antitumor immune responses and retarded tumor growth, which also showed good synergy with programmed cell death protein 1 (PD-1) antibody therapy. Overall, this study revealed a non-canonical role of HBx in driving HCC progression while offering potential anti-HCC immunotherapeutic strategies.

Hepatitis B virus (HBV) infection is a major threat worldwide and widely accepted as a critical risk factor of hepatocellular carcinoma (HCC), which accounts for almost 50% of the total new HCC cases every year (1, 2). HBV can insert its viral DNA into the host genome, which may promote oncogenic transformation of hepatocytes through multiple mechanisms, including induction of somatic mutations, genomic instability and persistent production of viral proteins (3). HBV possesses a 3.2 kb DNA genome that encodes the core protein, viral polymerase, envelope protein, and the X protein (HBx). Among the various HBV-encoding proteins, HBx has attracted particular interest in the context of HCC research. HBx plays an indispensable role in maintaining HBV replication, which has demonstrated significant relevance in HCC progression through modulating cell fate, proliferation, DNA repair and drug resistance (4, 5). For instance, HBx promotes HCC growth by activating the HSPA8-SLC7A11 axis to inhibit ferroptosis or inactivating p53 to inhibit apoptosis (6, 7). Current studies are mostly involved with the regulatory role of HBx in viral transcription and oncogenic transformation of hepatocytes, while its correlation with the immune microenvironment is rarely touched.

The persistent inflammation after HBV infection is widely accepted as the major driver of HCC pathogenesis (8, 9). During chronic HBV infection, multiple proinflammatory cytokines (interleukin (IL)-6, IL-1β and tumor necrosis factor) are produced by hepatocytes or tumor-associated macrophages (TAMs) to promote HCC progression (5, 10, 11). Notably, the pathological inflammation further forms a positive feedback loop to enhance the polarization of TAMs into tumor-promoting M2 type macrophages, leading to progressive HCC development and immunoresistance (12, 13). For instance, HBV stimulates IL-6 production and secretion into HCC TME (14), which subsequently promotes M2-like polarization of TAMs through activating IL-6 receptor (R)-signal transducer and activator of transcription STAT3 signaling axis for enhancing immunosuppression in HCC TME (15). Recent studies demonstrate IL-6/STAT3 axis is a potential immunotherapeutic target for reversing tumor immune evasion across multiple cancer types (16, 17, 18, 19). However, the correlation between HBV infection hallmarks such as aberrant HBx upregulation and TAMs in HCC remains elusive.

Hepatoma cells are widely recognized as the primary sites of viral infection and replication in HBV-induced HCCs. Based on our observations that patient-derived HBx-positive HCC samples prevalently showed higher CD206^+^ TAMs infiltration than their HBx-negative counterparts, we speculated that HBx-expressing HCC cells regulate polarization of TAMs. Of note, we found that HBx-expressing HCC cells promoted M2-like macrophage polarization *via* activation of macrophage-intrinsic IL-6-STAT3 signaling. The underlying mechanism is that the aberrant HBx upregulation markedly stimulated voltage-dependent anion channel (VDAC) 3 in HCC cells, causing mitochondrion DNA (mtDNA) release, thereby activating absent in melanoma 2 (AIM2)-interleukin (IL) 1β-nuclear factor kappa-B (NF-κB) axis in HCC cells to promote IL-6 secretion into TME. Meanwhile, IL-6 blockade readily reprogrammed M2-like TAMs into an immunosupportive M1-like phenotype, which potently strengthened the antitumor efficacy of programmed cell death protein 1 (PD-1) immune checkpoint blockade therapy. These findings reveal an underexplored role of HBx in promoting HCC development through contributing to the establishment and maintenance of the immunosuppressive TME, highlighting the potential utility of IL-6 blockade as an axillary treatment modality for amplifying the HCC responses to immune checkpoint blockade therapy.

## Results

### HBx-expressing HCC cells stimulate M2 polarization of TAMs

**Figure 1:**
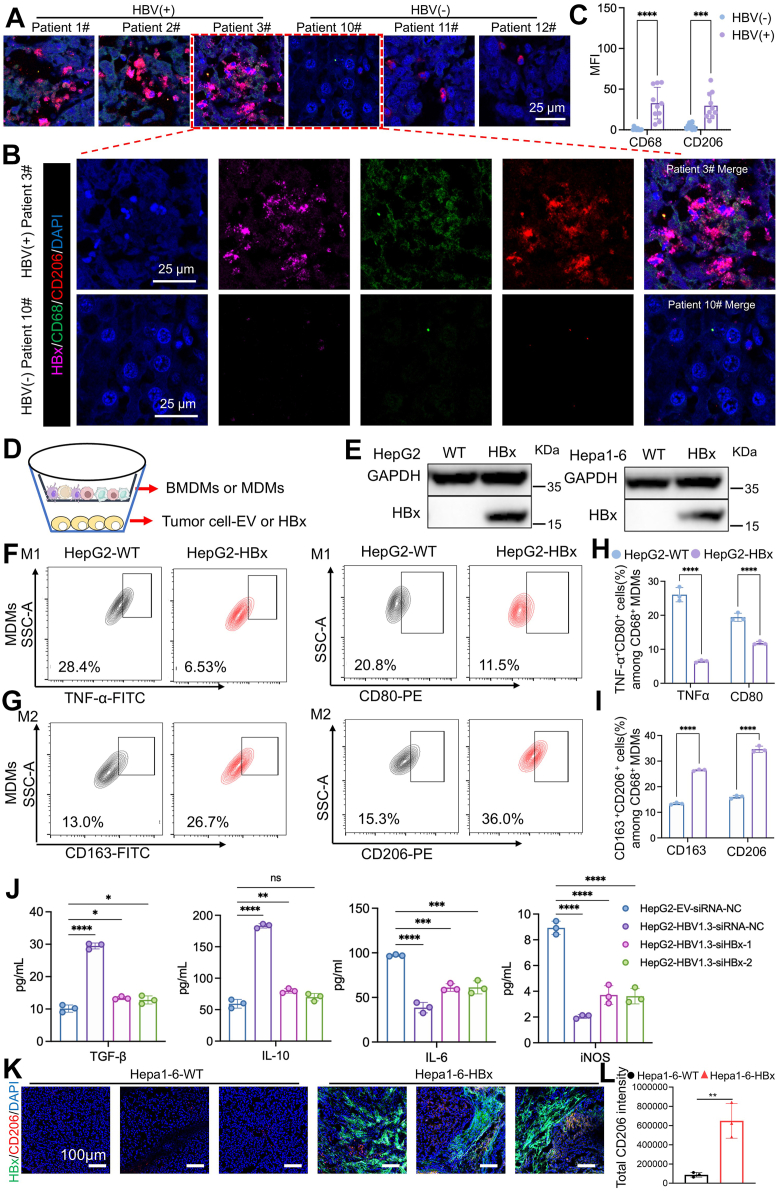
**HBx-positive HCC cells stimulate M2 polarization.***A* and *B*, representative images of CD68 and CD206 staining in patient-derived tissue samples from HBV (−) or HBV (+) HCC patients. HBx, *purple*; CD68, *green*; CD206, *red*; and nuclear staining (4′,6-diamidino-2-phenylindole, DAPI), *blue*. Scale bar: 25 μm. *C*, mean fluorescence intensity (MFI) of CD68 and CD206 expression from HBV(+) (n = 10) and HBV(−) (n = 10) HCC tumors, quantified using Image J. *D*, scheme of tumor-immune cell co-culture assays. *E*, WB detection of HBx and glyceraldehyde-3-phosphate dehydrogenase (GAPDH) in WT and HBx-positive HepG2 or Hepa1-6 cells. *F–I*, frequency of M1 type MDMs (TNFα^+^ or CD80^+^ cells) or M2 type MDMs (CD163^+^ or CD206^+^ cells) after being co-cultured with HepG2-WT cells or HepG2-HBx cells (*F* and *G*). Changes of percentages of TNFα^+^ or CD80^+^ cells among CD68^+^ M1 TAMs (*H*), CD163^+^ or CD206^+^ cells among CD68^+^ M2 TAMs (*I*). *J*, Enzyme-linked immunosorbent assay (ELISA) evaluation of TGF-β, IL-10, or IL-6 secretion, as well as iNOS expression in MDMs treated with the supernatants (Sup) from HBV1.3-transfected HepG2 with *HBx* knockdown or not (n = 3). *K* and *L*, representative images of CD206 staining in murine tumor tissues. HBx, *green*; CD206, *red*; and nuclear staining (DAPI). The scale bar represents 100 μm (*K*). MFI of CD206 expression in the murine tumor tissues (n = 3) (*L*). Statistical analysis was carried out using two-way-ANOVA. ns, no significance, ∗*p* < 0.05, ∗∗*p* < 0.01, ∗∗∗*p* < 0.001, ∗∗∗∗*p* < 0.0001. Error bars represent mean ± SD.

HBx is known to drive HCC progression through manipulating TAM activity, but the precise mechanisms remain to be elucidated (20, 21, 22). To delineate the interplay between HBx and TAMs, we initially performed immunofluorescence (IF) analysis to examine HBx expression and M2-TAM infiltration in HCC specimens from HBV-positive or -negative patients. The results indicated that CD68^+^CD206^+^ TAMs were obviously enriched within the HBx-positive regions of HCC samples (Figs. 1, *A*–*C* and S1*A*). HBV shows a strong tropism for HCC cells according to our observation and previous reports, indicating a strong correlation between HBx-producing HCC cells and TAM polarization (23). Extending from these preliminary findings, we constructed HBx-stable expressing HepG2 (a human hepatoblastoma-derived cell line) and HBx-Hepa1-6 (a murine HCC cell line), and then co-cultured those tumor cells with human primary monocyte-derived macrophages (MDMs) or mice bone marrow-derived macrophages (BMDMs) (Fig. 1, *D* and *E*). As expected, HCC-intrinsic HBx expression markedly enhanced M2 polarization of macrophages with elevated expression of CD206, CD163, transforming growth factor-β (TGF-β) and IL-10, accompanied by reduced expression of CD80, IL-6 and inducible nitric oxide synthase (iNOS) (Figs. 1, *F*–*I* and S1, *B–D*). In addition, we extracted murine splenetic immune cells for co-culturing assays. Hepa1-6-HBx cells markedly induced macrophage polarization to the M2 phenotype, while decreasing the population of interferon (IFN)-γ^+^CD8^+^ T cells, without affecting dendritic cell (DC) maturation (Fig. S1*E*). To further explore the effects of HBx on macrophage polarization during HBV infection, we first transfected HCC cells with plasmids containing a 1.3-fold-overlength HBV genome (HBV 1.3) to mimic HBV infection and then with si*HBx* (Fig. S2, *A* and *B*), afterwards the transfected HCC cells were co-incubated with BMDMs to investigate their immunoregulatory impact. Consistently, HBV-infected HCC cells drove M2 polarization in BMDMs in their co-culture assays, while those *HBx*-KD counterparts lost their capacity to drive M2-like polarization of macrophages (Figs. 1*J* and S2*C*). Moreover, we established subcutaneous Hepa1-6-WT or Hepa1-6-HBx tumor-bearing mouse models, and found the infiltration of F4/80^+^CD206^+^ TAMs in Hepa-1-6-HBx tumors was evidently higher than that in Hepa-1-6-WT group (Fig. 1, *K* and *L*). These data collectively illustrated that HBx remarkably induced tumoral infiltration of M2-like TAMs.

### HBx induces M2 polarization through triggering AIM2 activation

**Figure 2:**
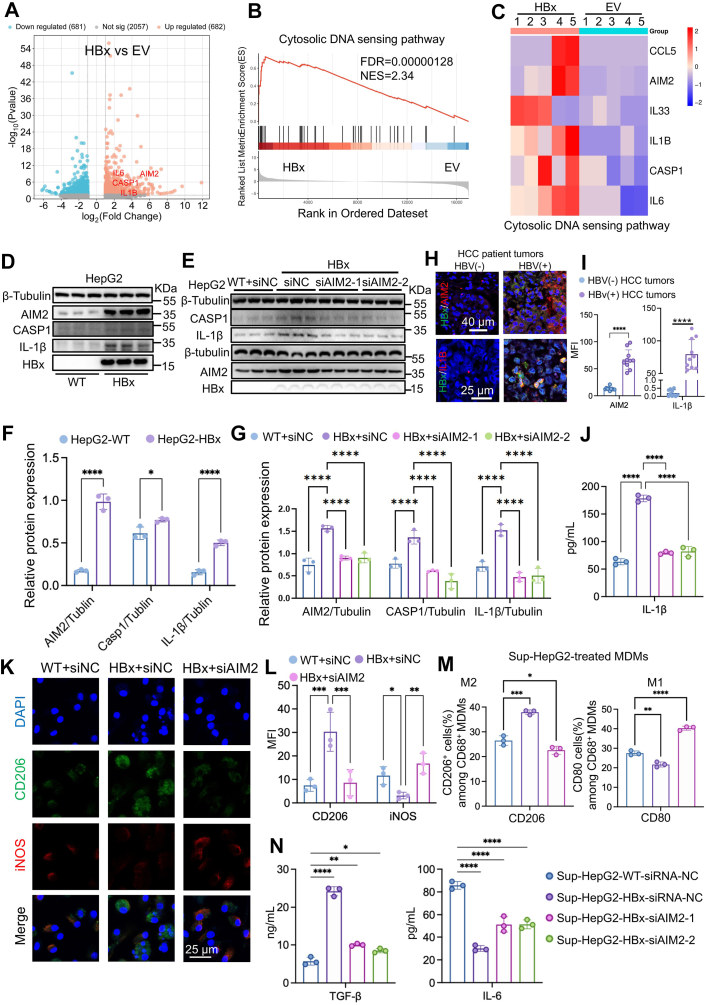
**HBx triggers AIM2 activation accounting for M2 polarization.***A*, Volcano plots showing DEGs. *B*, GSEA of the transcriptional signature about cytosolic DNA sensing pathway from HepG2 cells. *C*, heatmap of DEGs in cytosolic DNA sensing pathway from HepG2 cells. *D–G*, WB detection of AIM2, IL-1β, pro-caspase1 (CASP1), HBx and GAPDH in WT and HBx-expressing HepG2 with *AIM2* knockdown or not (*D* and *E*). Protein expression of AIM2, IL-1β, or CASP1 relative to GAPDH (n = 3) (*F* and *G*). *H* and *I*, representative images of HBx (*Green*) AIM2 (*Red*, *upper panel*) and IL-1β (*Red*, *lower panel*) staining in patient-derived tissue samples from HBV (−) or HBV (+) HCC patients. Scale bar: 25 μm (*H*). MFI of AIM2 and IL-1β expression from HBV(+) (n = 10) and HBV(−) (n = 10) HCC tumors (*I*). *J*, ELISA detection of IL-1β in HepG2 cells with different treatments. *K* and *L*, representative images of CD206 and iNOS staining in MDMs treated with the Sup from control or *AIM2* knockdown HepG2 cells. CD206, *green*; iNOS, *red*; and DAPI staining for nucleus. The scale bar represents 25 μm (*K*). MFI of CD206 or iNOS expression in HepG2 cells with the indicated treatments (*L*). *M*, changes in percentages of CD206^+^ cells among CD68^+^ M2 TAMs or CD80^+^ cells among CD68^+^ M1. *N*, ELISA detection of TGF-β or IL-6 production in MDMs treated with the Sup from control or *AIM2* knockdown HepG2 cells. Statistical analysis was carried out using one-way-ANOVA or two-way-ANOVA. ns, no significance, ∗*p* < 0.05, ∗∗*p* < 0.01, ∗∗∗*p* < 0.001, ∗∗∗∗*p* < 0.0001. Error bars represent mean ± SD.

**Figure 3:**
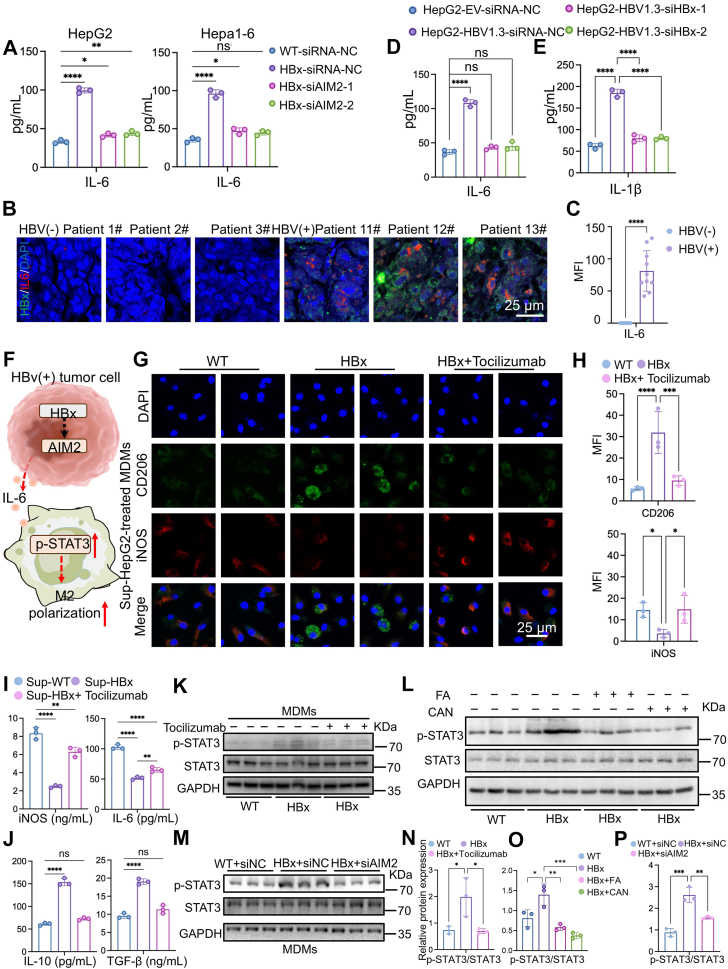
**HBx triggers tumor-intrinsic AIM2-IL-6 activation for promoting M2 polarization by activating macrophage-intrinsic STAT3 signaling.***A*, ELISA detection of IL-6 secretion in the Sup from control or *AIM2* knockdown cells. *B* and *C*, representative images of IL-6 and HBx staining in the clinical samples from HBV (−) or HBV (+) HCC patients. HBx, *green*; IL-6, *red*; and DAPI staining for nuclei, *blue*. The scale bar represents 25 μm (*B*). MFI of IL-6 expression from HBV(+) (n = 10) and HBV(−) (n = 10) HCC tumors (*C*). *D* and *E*, ELISA detection of IL-6 (*D*) or IL-1β (*E*) from the Sup of HBV 1.3 plasmid transfected-HepG2 cells with *HBx* knockdown or not. *F*, schematic illustration of IL-6-pSTAT3 axis for M2 macrophage polarization. *G* and *H*, representative images of CD206 or iNOS staining in MDMs treated with the Sup from HepG2-WT or HepG2-HBx cells in the presence or absence of Tocilizumab. CD206, *green*; iNOS, *red*; and DAPI staining for nuclei. The scale bar represents 25 μm (*G*). MFI of CD206 and iNOS expression in HepG2 cells with separate treatments (*H*). *I* and *J*, ELISA detection of TGF-β, IL-10, IL-6, or iNOS production in MDMs treated with the Sup from the indicated tumor cells in the presence of Tocilizumab or not. *K* and *L*, WB analysis of p-STAT3, STAT3 or GAPDH abundance from MDMs treated with the Sup from the indicated tumor cells in the presence of Tocilizumab (*K*), FA, CAN (*L*) or not. *M*, WB analysis of p-STAT3, STAT3 or GAPDH abundance from MDMs treated with the Sup from control or *AIM2* knockdown HepG2 cells. *N–P,* Protein expression of p-STAT3 relative to STAT3 (n = 3) from HepG2 cells with the indicated treatment. Statistical analysis was carried out using one-way-ANOVA or two-way-ANOVA. ns, no significance, ∗*p* < 0.05, ∗∗*p* < 0.01, ∗∗∗*p* < 0.001, ∗∗∗∗*p* < 0.0001. Error bars represent mean ± SD.

We next explored the mechanism of HBx-induced M2 polarization. We performed RNA-sequencing (RNA-seq) to analyze the gene expression patterns of HepG2-HBx and HepG2-WT cells. Our data showed that 682 genes were upregulated, while 681 genes were downregulated in HepG2-HBx cells *versus* HepG2-WT cells (absolute fold-change threshold >2, *p* < 0.05) (Fig. 2*A*). The raw gene expression data were then applied for Kyoto Encyclopedia of Genes and Genomes (KEGG) analysis to identify HBx-related pathways. We noticed that HBx-associated differentially expressed genes (DEGs) were significantly enriched in four signaling pathways, including cytokine-cytokine receptor interaction, Neuroactive ligand-receptor pathway, JAK-STAT signaling pathway and NF-κB signaling pathway (Fig. S3*A*). Gene Set Enrichment Analysis (GSEA) analysis was further conducted with KEGG gene sets, highlighting significant enrichment of DEGs (CCL*5*, *AIM2*, *IL-33*, *IL-6*, *CASP1*, and *IL1B*) in the cytosolic DNA-sensing pathway (NES = 2.34, *FDR* = 0.00000128) (Fig. 2, *A*–*C*). The raw sequence data can be downloaded from SRA database using PRJNA1420944 BioProject accession number. As expected, HBx markedly induced the upregulation of AIM2, IL-1β, and pro-caspase1 (CASP1) in tumor cells, but not in si*AIM2*-transfected ones (Figs. 2, *D*–*G* and S3, *B–E*). Enzyme-linked immunosorbent assay (ELISA) of collected cell culture supernatants further confirmed that IL-1β production was significantly increased in HBx-expressing HepG2 cells compared with WT HepG2 cells, and this effect was dependent on AIM2 expression (Fig. 2*J*). Consistently, both AIM2 and IL-1β levels were markedly elevated in tumor tissues from HBV-positive HCC patients relative to HBV-negative counterparts (Figs. 2, *H* and *I* and S4). To further highlight the role of HBx for HBV-induced IL-1β production, we transfected HBV 1.3 plasmid to HepG2 cells with *HBx* knockdown or not. The ELISA data showed that HBx was responsible for IL-1β secretion during HBV infection (Fig. 3*E*). Meanwhile, we examined the activation of cyclic GMP-AMP synthase (cGAS)-stimulator of interferon genes (STING) axis in HBx-expressing cells, which is another classical cytosolic DNA sensing pathway (24, 25). Consistent with the findings in previous studies (26), HBx expression was negatively correlated with the abundance of cGAS and phosphorylated STING while showing negligible impact on STING abundance (Fig. S5*A*), suggesting that HBx activated AIM2-mediated cytosolic DNA-sensing pathway in HCC cells.

We further explored the role of HCC-intrinsic AIM2 pathway in HBx-induced M2 macrophage polarization. After carrying out si*AIM2* transfection in HepG2-HBx cells, we collected the corresponding Sup to treat M0-like BMDMs. Notably, samples from the *AIM2*-KD group failed to induce obvious polarization of BMDMs towards an M2-like phenotype (Fig. 2, K-N, Fig S5B-D). Moreover, AIM2 expression showed positive correlation with the infiltration levels of M2-like TAMs in HCC tissues according to The Cancer Genome Atlas (TCGA) cohorts of hepatocellular carcinoma using TIMER2.0 (http://timer.cistrome.org/) (27) (Fig. S6). These data collectively suggested that HBx-mediated AIM2 activation drove M2 polarization of TAMs in HBV-related HCC.

### HBx promotes AIM2-associated IL-6 secretion and M2 macrophage polarization

Subsequently, we investigated how cancer cell-intrinsic HBx-AIM2 axis affected macrophage polarization. We noticed that IL-6, a crucial inducer of M2 polarization, was significantly induced in HBx-stable expressing cells, but not in *AIM2* knockdown cells (Figs. 3*A* and S7, *A* and *B*). We also detected abundant IL-6 expression within the HBx-positive regions in samples from HBV^+^HCC patients (Figs. 3, *B* and *C*, S7*C*). In addition, HBx was found predominantly responsible for IL-6 secretion in HBV 1.3 plasmid-transfected tumor cells (Fig. 3*D*). These results suggested that HBx-expressing HCC cells may promote M2 macrophage polarization in association with AIM2-IL-6 activation (Fig. 3*F*). To further explore the potential mechanism linking IL-6 secretion by HBx-expressing HCC cells with M2-like macrophage polarization, we generated HepG2-HBx-conditioned culture media to treat MDMs with or without co-treatment of the IL-6R inhibitor Tocilizumab. As expected, the Sup + Tocilizumab group showed negligible changes in the expression levels of several M2 markers (IL-10, TGF-β, and CD206) as well as M1 markers (IL-6 and iNOS) in MDMs (Figs. 3, *G*–*J* and S7, *D* and *E*). In addition, nitrite levels in culture supernatants were assessed using the Griess Reagent System as an indicator of NO production and showed a pattern consistent with the iNOS expression results (Fig. S7*F*). Interestingly, IL-1β treatment alone did not induce M2 polarization or affect the M2 polarization-promoting function of IL-6 (Fig. S8*A*). Considering that IL-6 induces M2 macrophage polarization *via* activating STAT3 (Figs. 3*F* and S8*B*), we also examined STAT3 phosphorylation in MDMs incubated with the indicated conditioned culture media. HepG2-HBx conditioned culture media induced obvious STAT3 phosphorylation in MDMs but not in those treated with Fructose-arginine (FA, an AIM2 inhibitor), or Canakinumab (CAN, an IL-1β inhibitor) (Fig. 3*L*). This attenuation may be attributable to the reduced IL-6 levels in the conditioned medium following FA or CAN treatment (Fig. S8*C*). Additionally, Sup from *AIM2* knockdown or FA-treated cells failed to activate the macrophage-intrinsic STAT3 signaling (Fig. 3, *K–P*). Together, these results support a model in which HBx promotes tumor-intrinsic IL-6 secretion in association with AIM2 signaling, thereby contributing to STAT3 activation and M2 macrophage polarization.

### HBx promotes IL-6 secretion in association with AIM2–IL-1β–NF-κB signaling

**Figure 4:**
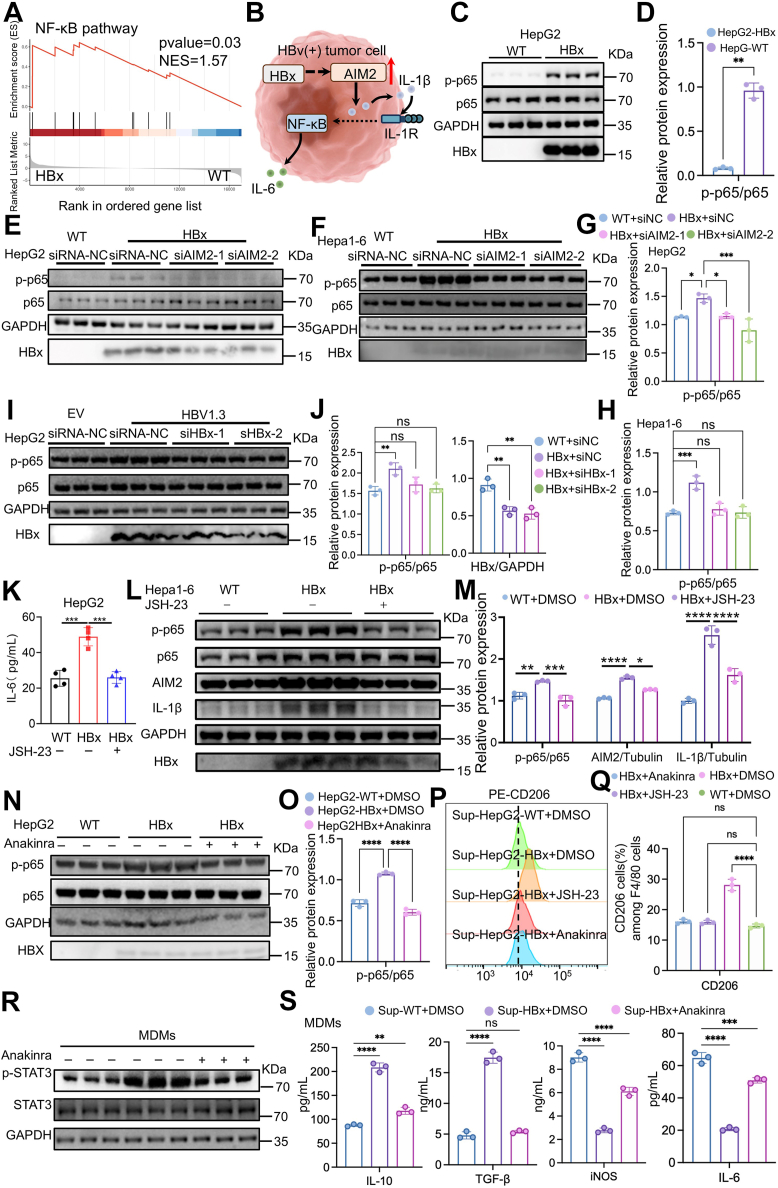
**HBx induces IL-6 secretion through activating AIM2-IL-1β-NF-κB signaling axis.***A*, GSEA of the transcriptional signature about NF-κB pathway. *B*, Schematic illustration of HBx-induced activation of AIM2-IL-1β-NF-κB-IL-6 axis. *C* and *D*, WB analysis of abundance of p-p65, p65, HBx or GAPDH in WT and HBx-related HepG2 (*C*). Protein expression of p-p65 relative to p65 in those HepG2 cells (*D*). *E–H*, Western blotting (WB) detection of p-p65, p65, HBx or GAPDH expression in control or *AIM2*-knockdown HepG2 (*E*) or Hepa1-6 (*F*) cells. Protein expression of p-p65 relative to p65 in those treated cells (*G* and *H*). *I* and *J*, WB analysis of expression of p-p65, p65, HBx or GAPDH in HBV 1.3 plasmid transfected-HepG2 cells with *HBx* knockdown or not (*I*). Protein expression of p-p65 relative to p65, and HBx relative to GAPDH in those treated cells (*J*). *K*, ELISA detection of IL-6 from the Sup of HepG2-WT and HepG2-HBx cells in the presence of JSH-23 or not. *L* and *M*, WB detection of p-p65, AIM2, IL-β, HBx or GAPDH expression in WT or HBX-related HepG2 cells treated with DMSO or JSH-23 (*L*). Protein expression of p-p65 relative to p65, AIM2 and IL-β relative to GAPDH in those treated cells (*M*). *N* and *O*, WB examination of expression of p-p65, p65, HBx or GAPDH in HepG2 cells in the presence of Anakinra or not (*N*). Protein expression of p-p65 relative to p65 in those treated cells (*O*). *P* and *Q*, flow cytometry analysis of CD206 expression in HepG2-WT and HepG2-HBx cells treated with DMSO, JSH-23, or Anakinra. *R*, WB examination of abundance of p-STAT3, STAT3 or GAPDH in MDMs treated with the Sup from HepG2-WT or HepG2-HBx in the presence of Anakinra or not. *S*, ELISA detection of TGF-β, IL-10, IL-6, or iNOS production in MDMs treated with the Sup from the indicated tumor cells in the presence of Anakinra or not. Statistical analysis was carried out using one-way-ANOVA, or two-way-ANOVA. ∗ ns, no significance, ∗*p* < 0.05, ∗∗*p* < 0.01, ∗∗∗*p* < 0.001, ∗∗∗∗*p* < 0.0001. Error bars represent mean ± SD.

We further explored the potential mechanism linking AIM2 signaling with IL-6 production in tumor cells. NF-κB is the master regulatory protein for IL-6 induction. Consistently, we found that DEGs were also enriched in the NF-κB pathway gene set from HepG2-HBx *versus* HepG2-WT RNA-seq data using GSEA analysis (Fig. 4*A*). It has been reported that IL-1β activates myeloid differentiation primary-response protein 88 (MyD88)-NF-κB-IL-6 axis through binding to IL1 receptor (IL1R) (28, 29, 30). We therefore hypothesized that HBx may promote IL-6 elevation in association with AIM2-IL-1β-IL1R-NF-κB signaling (Fig. 4*B*). HepG2-HBx and Hepa1-6-HBx cells showed obviously enhanced p65 phosphorylation (Figs. 4, *C*–*H* and S9*A*), while *AIM2* knockdown attenuated p65 activation in HCC cells (Fig. 4, *E*–*H*). Meanwhile, WB assay revealed that HBx upregulation drove p65 activation in HBV 1.3 plasmid-transfected tumor cells (Figs. 4, *I* and *J*, S9*B*). In addition, JSH-23 (an NF-κB inhibitor) (31) and Anakinra (an IL1R inhibitor) potently antagonized p65 phosphorylation and IL-6 secretion (Figs. 4, *K–O* and S9, *C* and *D*). However, p65 inhibition did not affect AIM2 and IL-1β expression in HBx-related HCC cells (Figs. 4, *L* and *M*, S9, *C* and *D*). These findings support the involvement of AIM2-IL-1β-associated signaling in NF-κB activation and IL-6 production in HBx-expressing tumor cells. We further collected the Sup from the HepG2-HBx cells with NF-κB or IL1R inhibition to treat MDMs, and found that JSH-23 or Anakinra-treated cells failed to increase the abundance of CD206 and phosphorylated STAT3 in MDMs (Fig. 4, *P–R*, and S9, *E–H*). ELISA and quantitative reverse transcription polymerase chain reaction (RT-qPCR) assay also verified that the Anakinra-treated tumor cells could not affect the expression of M2 markers (TGF-β, IL-10) and M1 markers (IL-6, iNOS) in MDMs (Figs. 4*S*, S9*I*). Consistently, nitrite levels in the culture supernatants showed no obvious changes under these conditions (Fig. S9*J*). Collectively, all these data support a model in which HBx-positive HCC cells promote M2 macrophage polarization in association with tumoral AIM2-IL-1β-NF-κB-IL-6 signaling.

### HBx activates AIM2 signaling pathway *via* causing mitochondria (mt) DNA leakage

**Figure 5:**
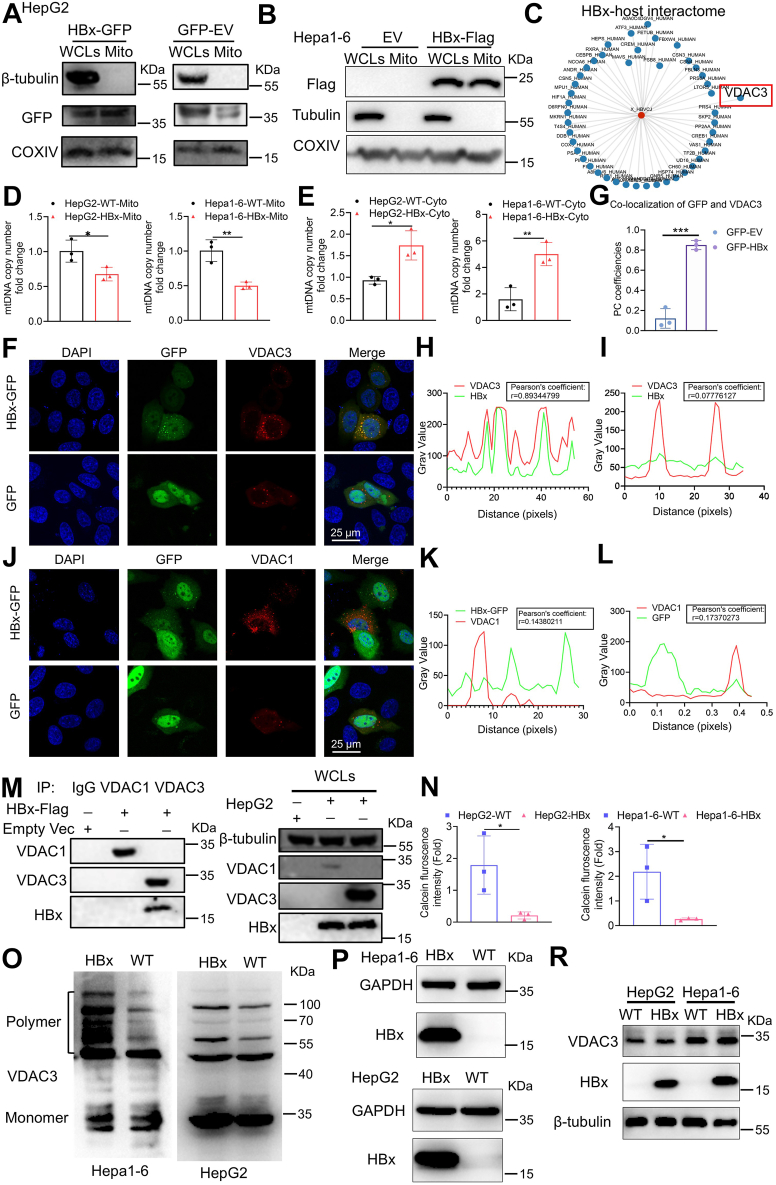
**HBx induces mtDNA release for AIM2 activation.***A* and *B*, WB examination of expression of HBx, COXIV or β-tubulin in whole cell lysates (WCLs) and mitochondrion (Mito) from HepG2 or Hepa1-6 cells with the enforced HBx expression. *C*, human host-HBv protein-protein interaction network. *D* and *E*, RT-qPCR analysis of the relative mtDNA copies in Mito or cytosols (Cyto) from WT or HBx-related tumor cells. *F–I*, representative images of HBx-GFP or GFP-EV and VDAC3 staining in HepG2 cells with the indicated transfection. GFP, *green*; VDAC3, *red*; and DAPI staining for nucleus. The scale bar represents 25 μm (*F*). Colocalization analysis of HBx or GFP and VDAC3 in those transfected cells using ImageJ (*G–I*). *J–L*, Representative images of HBx-GFP or GFP-EV and VDAC1 staining in HepG2 cells transfected with the indicated transfection. GFP, *green*; VDAC1, *red*; and DAPI staining for nucleus. The scale bar represents 25 μm (*J*). Colocalization analysis of HBx or GFP and VDAC1 in those transfected cells using ImageJ (*K* and *L*). *M*, WB detection of HBx, VDAC3, VDAC1 or β-tubulin expression in WCLs or the protein immunoprecipitants using anti-VDAC3 or VDAC1 antibody from HBx-Flag plasmid-transfected HepG2 cells. *N*, relative calcein fluorescence in WT or HBx-related tumor cells. *O*, WB detection of abundance of HBx, VDAC3 or β-tubulin in WT or HBx-related tumor cells. *P* and *Q*, WB analysis of HBx and GAPDH expression in WT or HBx-related tumor cells. *R*, WB analysis of VDAC3 monomer and polymer from WT or HBx-related tumor cells. Statistical analysis was carried out using one-way-ANOVA or Student’s *t* test. ∗*p* < 0.05, ∗∗*p* < 0.01, ∗∗∗∗*p* < 0.0001. Error bars represent mean ± SD.

Eventually, we explored the mechanism of HBx-induced AIM2 activation. HBx has been reported to mainly induce mtDNA damage in HCC cells (32). Therefore, it is reasonable to assume that HBx mainly affects mtDNA for AIM2 activation. To test this hypothesis, we transfected HBx-expressing plasmids into HepG2 and Hepa1-6 cells and examined whether HBx could relocate to mitochondria. WB analysis showed that HBx was partially translocated into mitochondria (Fig. 5, *A* and *B*). To investigate the mtDNA leakage, we extracted DNA from mitochondria or cytoplasm after removal of mitochondria in WT and HBx-related tumor cells, and conducted mtDNA analysis by qPCR. The results showed that HBx promoted the cytosolic accumulation of mtDNA (Fig. 5, *D* and *E*), thus enabling AIM2 activation (33, 34). We further dissected how HBx caused mtDNA leakage. After computational analysis of human-HBx-protein interactions through human–virus interaction database (HVIDB, http://zzdlab.com/hvidb/) (35), voltage-dependent anion channel (VDAC) 3 was screened at the intersection of HBx downstream signaling and mitochondrial function (Fig. 5*C*), which modulates mitochondrial outer membrane permeability for controlling the flow of nucleotides, metabolites, and ions (36), suggesting that HBx regulates VDAC3 for mtDNA release. The regulatory interaction of HBx on VDAC3 was further verified through immunoprecipitation (IP) and indirect immunofluorescence assay (IFA) (Figs. 5, *F–M*, S10, *A* and *B*). In contrast, neither GFP alone nor the control mitochondrial outer membrane protein VDAC1 exhibited appreciable interaction or co-localization with HBx, indicating a specific association between HBx and VDAC3 (Fig. 5, *F–M*). Notably, mitochondrial permeability transition pore (mPTP) opening and VDAC3 oligomerization-mediated macropores are suggested to mediate mtDNA release (37, 38). As expected, HBx expression induced evident, apoptosis-associated speck-like protein containing a CARD (ASC) speck formation (Fig. S10*C*), mPTP opening (Figs. 5*N* and S11) and VDAC3 oligomerization (Fig. 5*O*) in HCC cells without affecting VDAC3 abundance (Fig. 5, *P–R*). These results implied that mitochondrial HBx orchestrated the formation of VDAC3-dependent macropores to release mtDNA for AIM2 activation.

### HBx contributes to hepatocarcinogenesis by blocking antitumor immunity

**Figure 6:**
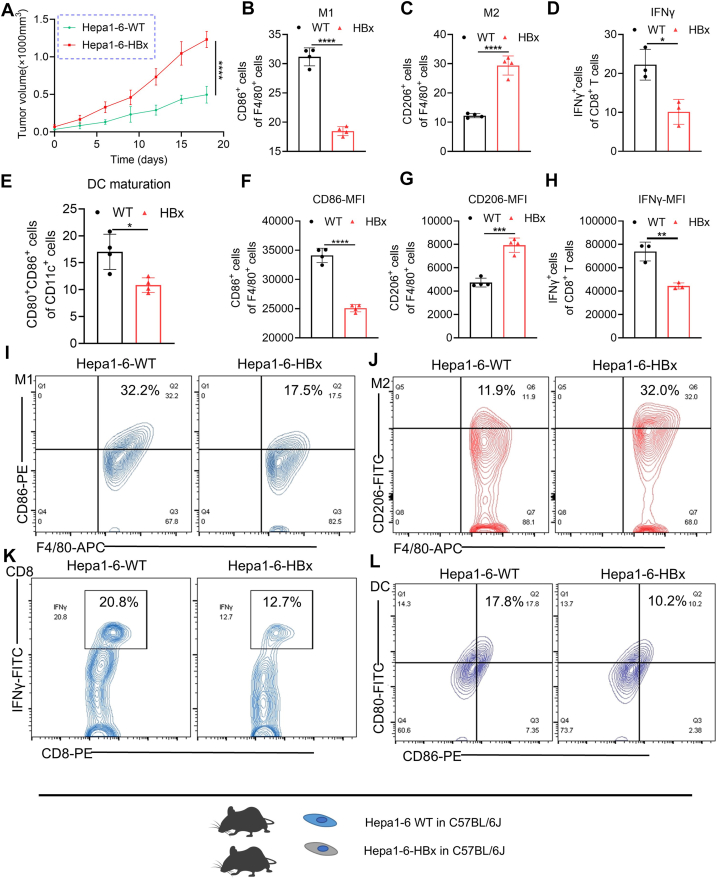
**HBx promotes murine tumor growth through inhibiting antitumor immunity.***A*, murine tumor growth curve. *B–E*, changes in percentages of CD86^+^ cells among F4/80^+^ M1 TAMs (*B*), CD206^+^ cells among F4/80^+^ M2 TAMs (*C*), IFNγ^+^ cells among CD8^+^ T cells (*D*), CD80^+^CD86^+^ cells among CD11c^+^ DCs (*E*) within murine tumors. *F–H* MFI indication of CD86 among M1 TAMs (*F*), CD206 among M2 TAMs (*G*), and IFNγ among CD8^+^ T cells. *I–L*, Flow cytometry assay (FCA) validation of M1 TAMs (*I*), M2 TAMs (*J*), CD8^+^ T cell activation (*K*), and DC maturation (*L*) from Hepa1-6-tumor bearing mice. Statistical analysis was carried out using Student’s *t* test. ∗*p* < 0.05, ∗∗*p* < 0.01, ∗∗∗*p* < 0.001, ∗∗∗∗*p* < 0.0001. Error bars represent mean ± SD.

To assess the role of HBx on HCC development, we established subcutaneous murine xenograft models using Hepa1-6-WT and Hepa1-6-HBx cells. HBx potently facilitated tumor development in immunocompetent mice (Fig. 6*A*), but not in immunodeficient nude mice (Fig. S14*A*), indicating the pro-tumorigenic roles of HBx through leveraging antitumor immunity. We further exacted the HCC tissues for flow cytometry analysis of tumor-associated immune cells. HBx-positive HCC tumors exhibited a typical immunosuppressive tumor environment (TIME) with reduced frequency of CD86^+^ M1 macrophages (Fig. 6, *B* and *I*), CD80^+^CD86^+^ DCs (Fig. 6, *E* and *L*), and IFNγ^+^CD8^+^ T cells (Fig. 6, *D* and *K*), as well as expanded population of CD206^+^ M2 macrophages (Fig. 6, *C* and *J*) comparing with that of HBx-negative tumors. MFI calculation consistently confirmed that HBx inhibited antitumor immune responses during HCC development (Fig. 6, *F*–*H*). Hence, these results illustrated that HBx expression in HCC cells promoted HCC growth through enhancing TAM polarization towards a pro-tumorigenic M2-like phenotype.

### IL-6 blockade boosts antitumor immunity and synergizes with anti-PD-1 therapy

**Figure 7:**
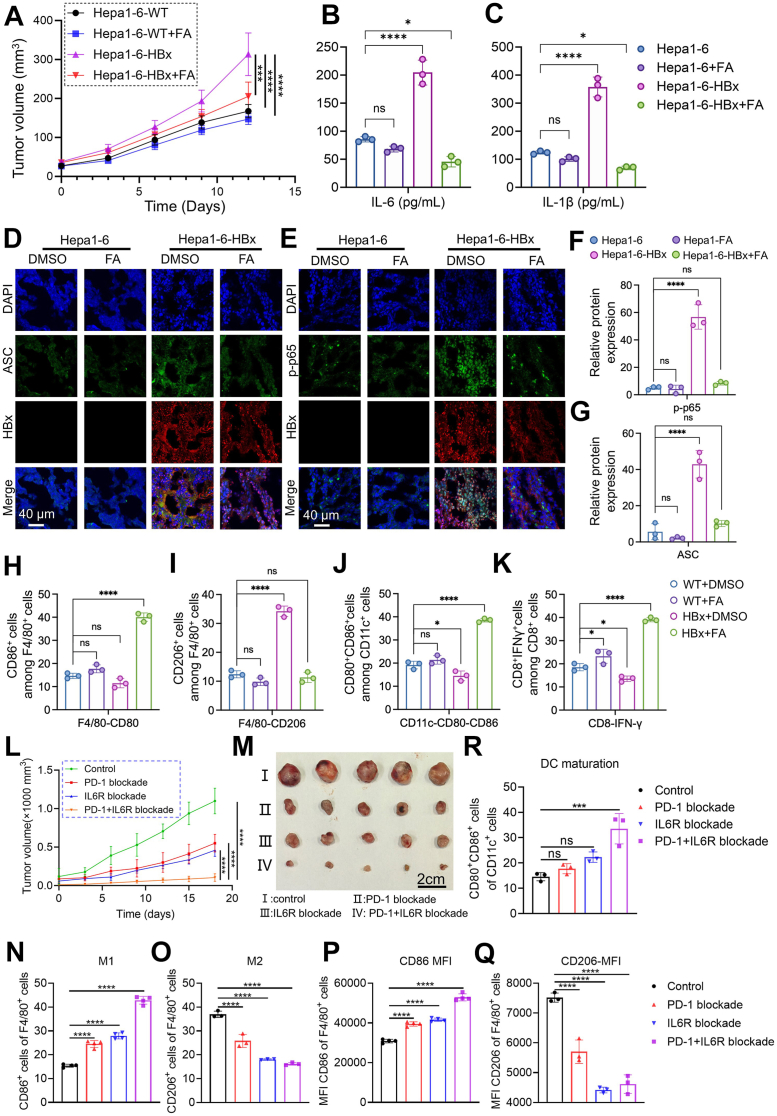
**IL-6 blockade potently triggers antitumor immune responses.***A*, tumor growth curves of Hepa1-6 tumor-bearing mice treated with FA, with the first day of administration defined as Day 0. *B* and *C*, ELISA examination of IL-6 and IL-1β production from the different murine tumor tissues. *D* and *E*, Representative images of HBx (*red*), ASC (*green*, *D*) or p-p65 (*green*, *E*) staining in murine tumor tissues with different treatments. The scale bar represents 25 μm. *F* and *G*, MFI of ASC (*F*) or p-p65 (*G*) expression in those tumors. *H–K*, Percentages of intratumoral CD86^+^ cells among F4/80^+^ M1 TAMs (*H*), CD206^+^ cells among F4/80^+^ M2 TAMs (*I*), CD80^+^CD86^+^ cells among CD11c^+^ DCs (*J*), and IFNγ^+^CD8^+^ T cells (*K*). *L* and *M*, changes in tumor growth and tumor size from Hepa1-6-tumour-bearing mice model. (*N*, *O* and *R*) Percentages of intratumoral, CD86^+^ cells among F4/80^+^ M1 TAMs (*N*), CD206^+^ cells among F4/80^+^ M2 TAMs (*O*), and CD80^+^CD86^+^ cells among CD11c^+^ DCs (*R*). *P* and *Q*, MFI indication of CD86 among M1 TAMs (*P*), and CD206 among M2 TAMs (*Q*) of Hepa1-6-tumor bearing mice treated with IL-6R Ab, PD-1 Ab or IL-6R Ab plus PD-1 Ab. Statistical analysis was carried out using one-way ANOVA. ns, no significance, ∗*p* < 0.05, ∗∗*p* < 0.01, ∗∗∗*p* < 0.001, ∗∗∗∗*p* < 0.0001. Error bars represent mean ± SD.

To evaluate HBx-triggered AIM2 signaling *in vivo*, we established Hepa1-6-HBx tumor-bearing mice treated with the AIM2 inhibitor FA. We observed that HBx expression drove the production of IL-6 and IL-1β and promoted ASC speck formation, thereby accelerating tumor growth (Figs 7, *A*–*G* and S12). In contrast, FA treatment significantly suppressed the secretion of these cytokines, inhibited ASC speck assembly, and restricted the growth of HBx-expressing tumors. Furthermore, FA enhanced antitumor immunity in HBx-driven tumors, primarily by inducing M2-to-M1 macrophage repolarization, promoting DC maturation, and activating cytotoxic CD8^+^ T cells (CD45^+^CD3^+^CD8^+^IFNγ^+^ cells, Figs. 7, *H*–*K* and S13). Moreover, to investigate if the HBx-stimulated IL-6 signaling could be therapeutically targeted for boosting HCC treatment efficacy, we established Hepa-1-6-HBx tumor bearing mice to assess the therapeutic potential of IL-6-blockade therapy through administering an IL-6R antibody (Ab). Tumor growth was substantially suppressed after IL-6R Ab treatment (Fig. 7, *L* and *M*). However, IL-6 blockade could not retard the development of HBx-negative tumors (Fig. S14*B*). In addition, we evaluated the stimulation of tumor-infiltrating immune cells with or without Tocilizumab. IL-6 blockade expanded the populations of M1 TAMs (CD45^+^F4/80^+^CD86^+^ cells) and cytotoxic CD8^+^ T cells, while suppressing M2 TAM populations (CD45^+^F4/80^+^CD206^+^ cells) (Figs. 7, *N–Q* and S15). Furthermore, the HCC-infiltrating DC population was not obviously affected by Tocilizumab treatment (Figs. 7*R* and S15).

HCC tends to demonstrate poor responses to immune checkpoint blockade therapy due to the complex TIME (39). Extending from our findings that IL-6 blockade could remodel the TIME, we evaluated the anti-tumor efficacy of IL-6 blockade + anti-PD-1 Ab combinational therapy in Hepa1-6-HBx tumor-bearing mice. The combination therapy achieved superior tumor suppression efficiency comparing to anti-PD-1 therapy or IL-6 blockade alone (Fig. 7, *L* and *M*). Also, combination therapy demonstrated superior immune activation efficacy including strengthened M2-M1 transition of TAMs, DC maturation and cytotoxic CD8^+^ T-cell responses, compared to monotherapy (Figs. 7, *N–R* and S15). These results collectively implied that IL-6 blockade in HBx-related tumors showed favorable synergy with PD-1 blockade therapy and triggered effective antitumor immune responses, thus providing a potential target for overcoming immunotherapy resistance of HBV^+^ HCC.

## Discussion

**Figure 8:**
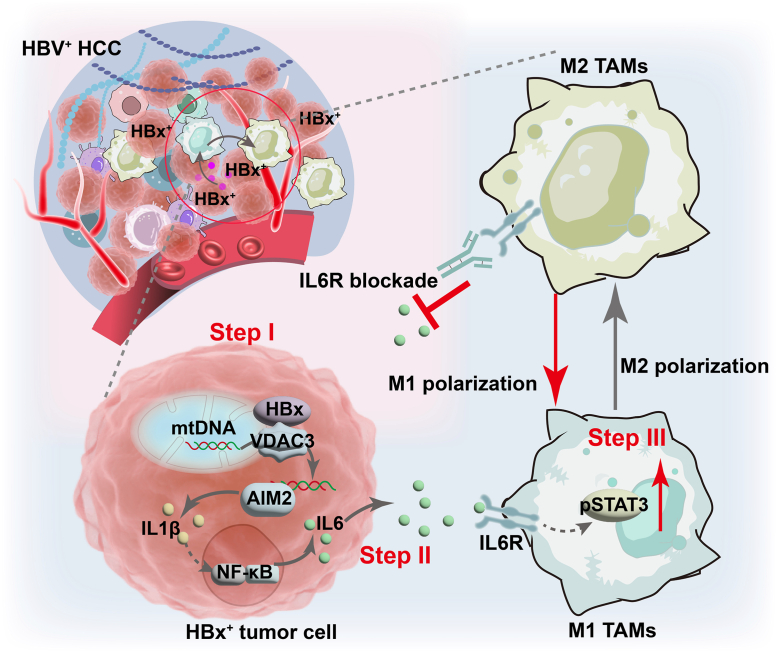
**Scheme illustration of mechanism of HBx-expressing tumor cells in inducing M2-like macrophage polarization.** During HBV-driven HCC development, HBx expression induces mtDNA release through interacting with VDAC3, accounting for AIM2-IL1β-NF-κB-IL-6 axis activation in tumor cell. The tumor-derived IL-6 further induces polarization of M2-like macrophage, which creates the immunosuppressive microenvironment. IL-6 blockade reverses the HBV-mediated immunosuppression, offering potential target for immunotherapy of HBV-related tumors.

Chronic HBV infection contributes to HCC development, attributed to its integration-caused persistent expression of viral proteins (40). As one of most important viral proteins, HBx plays multifunctional roles in promoting viral replication and hepatocarcinogenesis (5). Most of the current research focuses on its role in tumor initiation and metastasis through regulating host factors in tumor cells. However, HBx-immune microenvironment interaction in the context of HBV infection remains elusive. In the present study, we reported a non-canonical function of HBx in HCC development, which could promote HCC development by remodeling HCC-macrophage crosstalk to impair antitumor immunity (Fig. 8).

To our knowledge, there is only one report regarding the role of HBx in remodeling of HCC immune microenvironment through upregulating programmed cell death one ligand 1 (41). Although HBV can infect macrophages to influence their functions, hepatocytes are primary targets of HBV infection, which are frequently integrated with HBV genome. Therefore, it is reasonable to assume that tumor-intrinsic HBx expression could regulate TAM polarization to create TIME for HCC development. Here, we first collected HBV^+^ liver cancer tissues from real-life patients and detected abundant expression of HBx. CD206^+^ TAMs were enriched in HBx-related regions comparing to that in HBV^-^ counterparts. *In vitro* co-coculture assay and *in vivo* tumor models both indicated that HBx ^+^ HCC cells enhanced the infiltration of anti-inflammatory CD206^+^ M2 macrophages. We acknowledge that CD206^+^ TAMs exhibit functional heterogeneity, and that certain subsets have been reported to retain antigen-presenting capacity and even restrain tumor progression (42). To more rigorously evaluate M2-like polarization, we therefore assessed the expression of the canonical M2 marker CD163. Consistent with the CD206 findings, HBx-expressing tumor cells significantly increased the proportion of CD163^+^ MDMs, further substantiating a shift toward an immunosuppressive M2-like phenotype. We further explored how HBx-positive tumor cells enhanced M2 polarization. Based on RNA-seq analysis of WT and HBx-expressing tumor cells, we discovered significant enrichment of AIM2-IL-1β-NF-κB-IL-6 signaling axis in HBx^+^ HCC cells. Remarkably, HBx^+^ tumor cells showed a markedly reduced capacity to induce macrophage M2 polarization following IL-6 blockade or AIM2 inhibition, supporting the involvement of AIM2–IL-6-associated signaling in HBx-induced M2 macrophage polarization.

HBx subcellular localization significantly affects its virulent and pro-tumorigenic functions. For example, nucleus-localized HBx may enhance chromosomal instability or transcriptionally modulate the host factors for promoting HCC pathogenesis, progression and metastasis (43, 44, 45). Mitochondrion-localized HBx impairs the mitochondrial dynamics to support viral infection and inflammatory liver pathology while also triggering the leakage of mtDNA into cytosols (46, 47). Indeed, HBx upregulation is conducive for eliciting severe mitochondria damage, prompting us to investigate whether the post-HBV infection AIM2 activation was caused by the HBx-induced cytosolic DNA accumulation. As expected, HBx partially localized within mitochondria to cause mtDNA release into cytoplasm, during which HBx interacted with VDAC3 to trigger its polymerization and enforce mPTP opening for mtDNA leakage. Eventually, we identified that HBx disrupted mitochondrial membrane integrity to trigger mtDNA leakage for activating IL-6-dependent pro-tumorigenic HCC-macrophage crosstalk. As mitochondrial damage-associated signals are capable of activating multiple inflammasome pathways, the inflammatory consequences of HBx-induced mtDNA leakage may not be restricted to a single inflammasome subtype. Previous studies have implicated NLRP3 inflammasome signaling in HCC progression, while our current findings support the involvement of AIM2-associated signaling in HBx-driven IL-6-dependent immunomodulation. Considering the partially overlapping downstream inflammatory outputs of AIM2 and NLRP3 inflammasomes, further studies will be required to dissect the potential interplay and relative contribution of distinct inflammasome pathways in HBV-associated hepatocarcinogenesis.

Given the crucial role of IL-6 in TAM remodeling, an IL-6R inhibitor was exploited to treat HBx^-^ or HBx^+^ tumors *in vivo* for assessing the translational potential of immunotherapeutic concepts targeting the HBx-dependent immunosuppressive programs. Of note, IL-6 blockade effectively repolarized M2 TAMs into M1 TAMs in HBx-positive tumors and potently promoted the antitumor immune responses of PD-1 Ab therapy by stimulating DC maturation and CD8^+^ T cell activation. Combination of IL-6 blockade and PD-1 inhibitor elicited much superior tumor-suppressive effects than commercially available anti-PD-1 therapeutics, again validating the crucial role of HBx-stimulated pro-tumorigenic HCC-macrophage crosstalk in promoting HCC immune evasion as well as suggesting the therapeutic potential of IL-6 inhibitory modalities as a potent auxiliary treatment for boosting the anti-HCC efficacy of current immune checkpoint inhibitor therapies.

## Experimental procedures

### Subjects and specimen collection

All the patients were enrolled at the Second Affiliated Hospital of Army Medical University. These anonymized patients had not been treated with chemotherapy or radiation before tumor resection. The experiments and protocols in this study were approved by the Medical Ethics Committee of Second Affiliated Hospital of Army Medical University, Chongqing, China (Approval No.: #2022-406-01). The requirement for informed consent was waived by the Ethics Committee. Patients are considered HBV positive based on a history of chronic infection and HBV serological markers, and then classified into HBV-positive- and negative groups. Some of the fresh specimens, including normal and HCC tissues were used for immunofluorescence (IF) staining.

### Animal studies

All mouse experiments were carried out according to the guidelines of the Animal Care and Use Committee at the School of Medicine (Chongqing University). For tumor formation assay, cells were prepared in ice-cold PBS and injected subcutaneously (1 × 10^7^ cells) into BALB/c nude mice or C57BL/6J (female, 6 weeks old) mice. Tumor size was measured every 2 days using a vernier calliper, and tumor volumes were calculated by V = (a^2^ × b)/2, where a and b represent the shortest and longest tumor diameters, respectively. At 17 or 21 days after tumor cell injection, all animals were sacrificed to obtain tumors for IF staining.

### *In vivo* therapeutic treatment studies

For therapeutic studies, C57BL/6J mice were subcutaneously inoculated with Hepa1-6 or Hepa1-6-HBx cells in the right flank. Once tumors reached approximately 50 to 100 mm^3^, mice were randomly assigned to the indicated treatment groups. FA was administered intraperitoneally at a dose of 30 mg/kg once every two days for two consecutive weeks. For IL-6 blockade experiments, mice received anti-IL-6R antibody (clone MR16-1) intraperitoneally at 10 mg/kg twice weekly. For combination therapy experiments, anti-PD-1 antibody (clone RMP1-14; 200 μg per mouse) was administered intraperitoneally twice weekly together with anti-IL-6R antibody according to the same schedule. Control mice received equivalent volumes of vehicle or isotype control antibodies. At the experimental endpoint, mice were sacrificed and tumors were harvested for flow cytometric, histological, and molecular analyses.

### Cell lines

Tumor cell lines, including human hepatocellular carcinoma cell line (HepG2, RRID: CVCL_0027) and murine hepatoma cell line (Hepa1-6, RRID: CVCL_0327) were all purchased from ATCC and authenticated using short tandem repeat (STR) profiling upon arrival. All the cells were maintained in DMEM medium (Basal media technologies) with 10% fetal bovine serum (FBS, Basal media technologies, Shanghai, China) and penicillin-streptomycin (Solarbio). All cells were incubated in a humidified incubator with 5% CO_2_ at 37 °C. These cell lines were routinely screened for *mycoplasma* contamination using a PCR-based detection kit and regularly examined morphologically *via* optical microscopy to monitor the potential microbial (*e*.*g*., bacterial, fungal) contamination. Only microbial-free cell lines were used for experiments.

Splenic cells were obtained from healthy C57bL/6J mice. In brief, the spleens were extracted and ground in PBS using the flat end of the syringe. The splenic cells were then passed through 70 μm sterile Nylon strainer into the 100 mm culture dishes, followed by incubation with red blood cell lysis buffer for 2 min at room temperature (RT) to obtain the splenic lymphocytes. The lymphocytes were subjected to the co-culture assay.

### Reagents, plasmids and antibodies

The following reagents were used for *in vitro* experiments in this study: human recombinant M-CSF (MedChemExpress, MCE, #HY-P7050), murine recombinant M-CSF (MCE, #HY-P7085), recombinant Human IL-6 Protein (#90107ES08, YEASEN), recombinant Mouse IL-6 Protein (#90146ES05, YEASEN), Anakinra (#HY-108841, MCE), Tocilizumab (#HY-P9917, MCE), JSH-23 (#HY-13982, MCE), ethylene glycol-bis(succinic acid N-hydroxysuccinimide ester) (EGS, #21024ES60, YEASEN). pcDNA3.1-HBx-Flag and pcDNA3.1-HBx-GFP were synthesized by Sangon Biotech (Shanghai, China). The plasmid containing HBV 1.3-mer WT replicon (HBV 1.3) was purchased from (#V009181, NovoPro, Shanghai, China). Antibodies were listed in Table 1.

**Table 1:** Antibodies

Name	Supplier	Cat. No
cGAS	Proteintech	26416-1-AP
GAPDH	Proteintech	60004-1-Ig
AIM2	Proteintech	20590-1-AP
CASP1	Cell Signaling Technology	83383T
IL-1β	Proteintech	26048-1-AP
β-tubulin	Proteintech	10094-1-AP
HBx	Abcam	ab309352
p-STING	Cell Signaling Technology	19781
STING	Cell Signaling Technology	13647
p-p65	Cell Signaling Technology	3033
p65	Cell Signaling Technology	8242
p-STAT3	Cell Signaling Technology	9131
STAT3	Proteintech	10253-2-AP
VDAC3	Proteintech	55260-1-AP
VDAC1	Proteintech	10866-1-AP
ASC	Proteintech	10500-1-AP
anti-mouse CD45- pe/cy7	Biolegend	103,113
anti-mouse CD206-FITC	Elabscience	E-AB-F1135C
anti-human CD206-APC	Elabscience	E-AB-F1161E
anti-human CD206-FITC	Elabscience	E-AB-F1161C
anti-mouse F4/80-APC	Elabscience	E-AB-F0995E
anti-human F4/80	Santa Cruz	sc-52664
anti-human F4/80-APC	This paper	
anti-mouse-CD11c-APC	Elabscience	E-AB-F0991E
anti-mouse CD86-PE	Elabscience	E-AB-F0994D
anti-mouse CD80-FITC	Elabscience	E-AB-F0992C
anti-mouse CD8a-PE	Elabscience	E-AB-F1104D
anti-mouse CD3-APC	Elabscience	E-AB-F1013E
anti-mouse IFNγ-FITC	Elabscience	E-AB-F1101C
CoraLite®594-conjugated iNOS	Proteintech	CL594-18985
Alexa Fluor 488-conjugated goat anti-mouse IgG	Beyotime	A0428
HBx	Abcam	ab309352

### Preparation of BMDMs or MDMs

BMDMs was prepared as previously described (48). Briefly, hind legs from 6-8-week-old female C57BL/6j were used to extract bone marrows. The bone marrows were transferred into ice-cold PBS and filtered through the 80 μm cell strainer. After removal of red blood cells (RBC) using the RBC lysis buffer, the cell suspension was cultured into bone marrow culture medium with Dulbecco's Modified Eagle Medium (DMEM) supplemented with 1 ng/ml macrophage colony-stimulating factor 1 (M-CSF1), 10% fetal bovine serum (FBS), and 1% penicillin-streptomycin solution 10% heat-inactivated FBS, at 37 °C 5% CO_2_ incubator (Day 0). At Day 3, the bone marrow culture medium was replaced with the fresh one. BMDMs were subjected to the following experiments after 7 days’ culture. MDMs were prepared according to a previous study (49). In brief, peripheral blood mononuclear cells (PBMCs) were obtained from healthy donors, and then cultured in DMEM supplemented with 50 ng/ml recombinant human M-CSF, 10% FBS, and 1% penicillin-streptomycin solution, 10% heat-inactivated FBS. At Day 3, half of cell culture medium was replaced with fresh one. MDMs were harvested at Day 7 for the subsequent experiments.

### VDAC3 cross-linking assay

WT or HBx-related cells were washed and collected in PBS with incubation of the cross-linking reagent EGS (200 μm) for 40 min at 30 °C. Protein samples were subjected to sodium dodecyl sulfate polyacrylamide gel electrophoresis (SDS-PAGE) and WB analysis using the anti-VDAC3 antibody (RRID: AB_10973676).

### Confocal laser scanning microscope (CLSM)

Fresch HCC clinical or pre-clinical tissues were processed into the frozen section, which was incubated with mouse monoclonal antibody against F4/80 (RRID: AB_629466), and/or rabbit monoclonal antibody against HBx at 4 °C overnight. After being washed with phosphate-buffered saline (PBS) for 3 times, the frozen tissues were incubated with Alexa Fluor 488-conjugated goat anti-mouse IgG (RRID: AB_2893435) at RT for 30 min followed by staining with allophycocyanin (APC) anti-mouse CD206 antibody at RT for 60 min. After 4′,6-diamidino-2-phenylindole (DAPI) staining, the HCC samples were subjected to CLSM analysis. BMDMs or MDMs were fixed using 4% paraformaldehyde (PFA), followed by gentle washing with ice-cold PBS at RT for twice. Cells were then permeabilized with Saponin at RT for 10 min and gently washed by PBS for 3 times. After blocking with 5% bovine serum albumin (BSA)-PBS at RT for 60 min, cells were stained with FITC-conjugated CD206 antibody (RRID: AB_3065037), or CoraLite594-conjugated iNOS (RRID: AB_2919849) at 4 °C overnight. Cells were exploited to evaluate M1 or M2 polarization after nuclear staining with DAPI.

### Co-culture assay

BMDMs, MDMs, or splenic lymphocytes were seeded on the upper chamber of the Transwell, while different tumor cells were seeded in the lower chamber. After 24 h co-cultures, the immune cells were collected for FCA or RT-qPCR detection of M1 or M2 macrophage polarization.

### Mitochondrial extraction

Mitochondrial extraction was performed under the manufacturer’s instruction of Cell Mitochondria Isolation Kit (#C3601, Beyotime, Shanghai, China). In brief, cells were collected after being washed with ice-cold PBS. These cells were treated with Mitochondria Isolation Reagent and homogenized for 10-15 min. The cell homogenates were centrifuged at 600*g* for 10 min at 4 °C. The cell pellets contained the isolated mitochondria, and the supernatants contain the cytosolic components. Those components were applied for WB detection or qPCR analysis of mtDNA.

### DNA extraction

Differently treated cells were lysed using Radio Immunoprecipitation Assay (RIPA) lysis buffer (#P0013B, Beyotime) followed by removal of cell debris by centrifugation. Proteinase K was then used for proteolysis. After that, a mixture of phenol and chloroform (phenol/chloroform/isoamyl alcohol ratio is 25:24:1) was used for protein precipitation, followed by centrifugation and washing steps. RNase was exploited for RNA removal. The ice-cold ethanol was subsequently used for precipitation of DNA. The extracted DNA was redissolved in double-distilled water and kept in −80 °C.

### RT-qPCR

MDMs or BMDMs treated with IL-6 or supernatants of different tumor cells were harvested for RNA extraction with TRIzol, chloroform and isopropyl alcohol as previously described (50). The extracted RNAs were reverse transcribed into cDNA using UEIris RT mix with DNase under the manufacturer’s instruction. These cDNAs were subjected to RT-qPCR detection of mRNAs of human or murine IL-6, iNOS, IL-10, and TGF-β. The indicated primers were synthesized by Sangon Biotech (Table 2).

**Table 2:** qPCR primers

Gene name	Forward primer sequence (5′-3′)	Reverse primer sequence (5′-3′)
Mus TGFB1	CACCGGAGAGCCCTGGATA	TGTACAGCTGCCGCACACA
Mus iNOS	TCACCTTCGAGGGCAGCCGA	TCCGTGGCAAAGCGAGCCAG
Mus IL-10	CAGAGCCACATGCTCCTAGA	TGTCCAGCTGGTCCTTTGTT
Mus IL-6	TAGTCCTTCCTACCCCAATTTCC	TTGGTCCTTAGCCACTCCTTC
Mus β-actin	CGTTGACATCCGTAAAGACC	TAGGAGCCAGAGCAGTAATC
Human TGFB1	CTAATGGTGGAAACCCACAACG	TATCGCCAGGAATTGTTGCTG
Human iNOS	GCTCTACACCTCCAATGTGACC	CTGCCGAGATTTGAGCCTCATG
Human IL-10	CGCTAGAACCAAGCTGTCCT	CACATGCGCCTTGATGTCTG
Human IL-6	AGGGCACGAAGGCTCATCATT	AGGGCACGAAGGCTCATCATI
Human GAPDH	GTCTCCTCTGACTTCAACAGCG	ACCACCCTGTTGCTGTAGCCAA

### Western blotting (WB)

Differently treated tumor cells, BMDMs or MDMs were lysed using WB lysis buffer after being washed with PBS. The cell lysates were centrifuged at 4 °C for 15 min to remove the cell debris. The cell lysate supernatants were then subjected to SDS-PAGE followed by being transferred onto polyvinylidene fluoride (PVDF) membrane (#ISEQ07850, Sigma, Shanghai, China). The transferred PVDF membranes were blocked with 5% non-skimmed milk or bovine serum albumin in PBS containing 0.1% Tween 20 (PBST) at RT for 2 h, and subsequently incubated with primary antibodies targeting cGAS (RRID: AB_2880507), p-STING (RRID: AB_2737062), STING (RRID: AB_2732796), HBx, GAPDH (RRID: AB_2107436), IL-1β (RRID: AB_2880351), pro-caspase1 (CASP1), AIM2 (RRID: AB_10694420), p-STAT3 (RRID: AB_331586), STAT3 (RRID: AB_2302876), p-p65 (RRID: AB_331284), p65 (RRID: AB_10859369) or β-tubulin (RRID: AB_2210695) at 4 °C overnight. After being washed with PBST for 3 times, the PVDF membranes were incubated with HRP-labeled goat anti-rabbit IgG or HRP-labeled goat anti-mouse IgG at RT for 2 h. Protein signals were detected using ECL chemiluminescence kit on a ChemiDoc XRS gel imaging system.

### FCA

BMDMs, MDMs, intratumoral or splenic lymphocytes were incubated with Fc receptor blocking solution, and subsequently stained with 7-AAD viability staining solution for dead cell exclusion, and APC anti-mouse (or human) F4/80 antibody, FITC anti-mouse (or human) CD80 antibody for M1-like macrophage detection, or APC anti-mouse (or human) F4/80 antibody, FITC anti-mouse (or human) CD206 antibody for M2 macrophage detection. Intratumoral or splenic lymphocytes were stained with 7-AAD viability staining solution and PE/Cy7 anti-mouse CD45 (RRID: AB_312978) for immune cell gating. For IFN-γ analysis of CD8^+^ T cells, the cells were treated with Brefeldin A and then fixed with 4% PFA. After being permeabilized with Saponin, the cells were incubated with Fc receptor blocking solution at RT for 30 min. The cells were subjected to FCA after being stained with Zombie Violet Fixable Viability Kit, APC-anti-mouse CD3 (RRID: AB_3675272), PE-anti-mouse CD8a (RRID: AB_3065039) and FITC-anti-mouse IFN-γ (RRID: AB_3661692). For analysis of DC maturation, intratumoral or splenic lymphocytes were incubated with 7-AAD viability staining solution, pe/cy7 anti-mouse CD45, APC-anti mouse CD11c, FITC-anti mouse CD80 and PE-anti-mouse CD86 (RRID: AB_3102044) at RT for 15 min after Fc receptor blocking. All the data were analyzed using FlowJo software (RRID: SCR_008520) or CytExpert2.4 (RRID: SCR_017217).

### RNA-seq data processing

A total of 1 × 10^7^ HepG2-WT cells or HepG2-HBx cells were lysed with TRIzol Reagent for total RNA isolation. mRNAs were reverse transcribed into cDNA for subsequent RNA-seq library preparation (Sangon Biotech). After its quality inspection, the RNA-seq library was sequenced using Illumina Xten to obtain the general transcriptome sequencing data. The raw sequencing reads were subjected to quality control analysis and the filtered reads were then aligned against the human reference genome (hg19). Heatmaps and volcano plots of selected genes according to their functions were generated using https://www.bioinformatics.com.cn, an online platform for data analysis and visualization. Colors represent the log2 fold change values, which are calculated according to the log2-transformed gene transcript per million (TPM) value obtained from DESeq2 (RRID: SCR_015687). KEGG (http://www.genome.jp/kegg/, RRID: SCR_012773) pathway enrichment analysis was performed to determine the significantly enriched signal transduction pathways of host DEGs enriched in HepG2-HBx cells. GSEA (RRID: SCR_003199) was performed using the Molecular Signatures Database (MSigDB). Significance score of GSEA was calculated by the normalized enrichment score (NES) and false discovery rate (FDR). *p* < 0.05 indicated that KEGG pathways or GSEA pathways of the host DEGs were significantly enriched.

### RNA interference

siRNAs targeting HBx, murine AIM2, or human AIM2 were synthesized from Bsyntech. Those siRNAs were transfected into HepG2 (or Hepa1-6 cells) with or without HBV 1.3 plasmid transfection for 48 h in respective, and the *HBx* or *AIM2* knockdown cells were harvested for WB detection. The sequences of siRNAs were listed in Table 3.

**Table 3:** siRNA sequences

Gene name	siRNA sequence (5′-3′)
siRNA-mus AIM2-1	ACAUAGACACUGAGGGUAUTT
siRNA-mus AIM2-2	UGUCUAAGGCUUGGGAUAUTT
siRNA-mus AIM2-3	CUACCUGAGGAUAGCAUUUTT
siRNA-human AIM2-1	UAUGGUGCUAUGAACUCCAGAUGUCTT
siRNA-human AIM2-2	UUUCAGCUUGACUUAGUGGCUUUGGTT
siRNA-human AIM2-3	UUCUCUGAUAGAUUCCUGCUGGGCCTT
siRNA-HBx-1	GGUCUUACAUAAGAGGACUTT
siRNA-HBx-2	GGACGUCCUUUGUUUACGUTT
siRNA-HBx-3	CCGACCUUGAGGCAUACUUTT
siRNA-HBx-4	UGUGCACUUCGCUUCACCUTT

### Plasmid transfection

1 μg HBx-fused green fluorescent protein (GFP) expressing plasmid or GFP empty vector was transfected into HepG2 cells for 36 h. The transfected cells were then subjected to CLSM analysis of co-localization of HBx and VDAC3/or VDAC1.

### ELISA

IL-6 in the supernatants of cells with indicated treatments were detected using the commercial human or murine IL-6 ELISA kit (#CSB-E04638h or # CSB-E04639m, CUSABIO) under the manufacturer’s instruction. Human IL-1β, and TGF-β levels in cell culture supernatants were measured using the commercial human IL-1 beta ELISA Kit (#RK00001, Abclonal) and human TGF-β1 ELISA kit (#CSB-E04725h, CUSABIO) and according to the manufacturers’ instructions. iNOS was detected in the cell lysates with the specific treatments using the commercial human iNOS ELISA Kit (#ZC-35593, ZCI Bio).

### Measurement of nitric oxide (NO)

NO production was assessed by measuring nitrite (NO_2_^-^), a stable breakdown product of NO, in cell culture supernatants using the Griess Reagent System (#S0021S, Beyotime) according to the manufacturer’s instructions. Briefly, MDMs were first incubated with conditioned media derived from HepG2-WT or HepG2-HBx cells under the indicated treatment conditions, followed by stimulation with LPS/IFNγ to induce M1 polarization. Culture supernatants were then collected and incubated with sulfanilamide solution and N-1-naphthylethylenediamine dihydrochloride solution. Absorbance was measured at 540 nm, and nitrite concentrations were calculated using a sodium nitrite standard curve. Nitrite levels were used as an indicator of NO production and iNOS functional activity.

### Co-immunoprecipitation (co-IP) assay

Briefly, cells expressing HBx were lysed in IP lysis buffer. For each reaction, 500 μg of total protein lysate was incubated with 2 μg of the respective primary antibody (anti-VDAC1 or anti-VDAC3) or an equivalent amount of normal IgG (as a negative control) for 2 h at 4 °C with gentle rotation. Subsequently, 20 μl of pre-washed Protein A/G agarose beads were added to each sample, followed by incubation overnight at 4 °C with rotation. The beads were then collected by centrifugation and washed three times with ice-cold lysis buffer. The bound immune complexes were then eluted by boiling in 1 × SDS loading buffer for 10 min, and subsequently subjected to SDS-PAGE.

### Statistical analysis

All data were processed and analyzed using Graphpad Prism software (http://www.graphpad-prism.cn/, RRID:SCR_002798) by unpaired two-tailed *t* test, one-way-ANOVA without repeated measures or two-way-ANOVA. Bonferroni's multiple comparisons test or Dunnett's multiple comparisons test were used in one-way ANOVA and two-way ANOVA analysis. All experiments were repeated at least twice with three replicates involved. All data in Figures were shown as group mean and standard deviation (SD). The asterisks, as follows: ns, no significance; ∗, *p* < 0.05; ∗∗, *p* < 0.01; ∗∗∗, *p* < 0.001; ∗∗∗∗, *p* < 0.0001, indicate the statistical significance.

### Ethics committee approval and patient consent

This study was conducted at the School of Life Science, Chongqing University (Chongqing, China), and all animal experiments were performed according to the guidelines of the Animal Care and Use Committee at the School of Medicine (Chongqing University, Chongqing, China). The procedures for the use and analysis of human samples were approved by the Medical Ethics Committee of Second Affiliated Hospital of Army Medical University (Approval No.: #2022-406-01).

## Data availability

Data will be made available upon request.

## Supporting information

This article contains supporting information.

## Conflict of interest

The authors declare that they have no conflicts of interest with the contents of this article.
