# Supplementary material for: Hepatitis B virus promotes hepatocellular carcinogenesis by activating IL-6-dependent tumor-macrophage crosstalk and M2-like macrophage polarization
Source: J Biol Chem. 2026 Jun 26;302(8):113283. doi: 10.1016/j.jbc.2026.113283 (PMC13400354; doi:10.1016/j.jbc.2026.113283)
Supplement: Supplementary Material [file mmc1.docx]

**Hepatitis B virus promotes hepatocellular carcinogenesis by activating IL-6-dependent tumor-macrophage crosstalk and M2-like macrophage polarization**

Yingqi Liu^1#^, Xinyi Cao^1#^, Bingbing Yang^1#^, Hongmei Zheng^1^, Hongyu Zhong^1^, Dan Tao^2^, Menghuan Li^1*^, Zhong Luo^1*^

^1^School of Life Science, Chongqing University, Chongqing 400044, P. R. China.

^2^Department of Radiation Oncology, Chongqing University Cancer Hospital, Chongqing 400044, P. R. China.

^#^Yingqi Liu, Xinyi Cao and Bingbing Yang contributed equally to this work.

^*^Correspondence: Zhong Luo, School of Life Science, Chongqing University, Chongqing 400044, P. R. China. Email: luozhong918@cqu.edu.cn Menghuan Li, School of Life Science, Chongqing University, Chongqing 400044, P. R. China. Email: menghuanli@cqu.edu.cn

**Supplementary figures and figure captions**


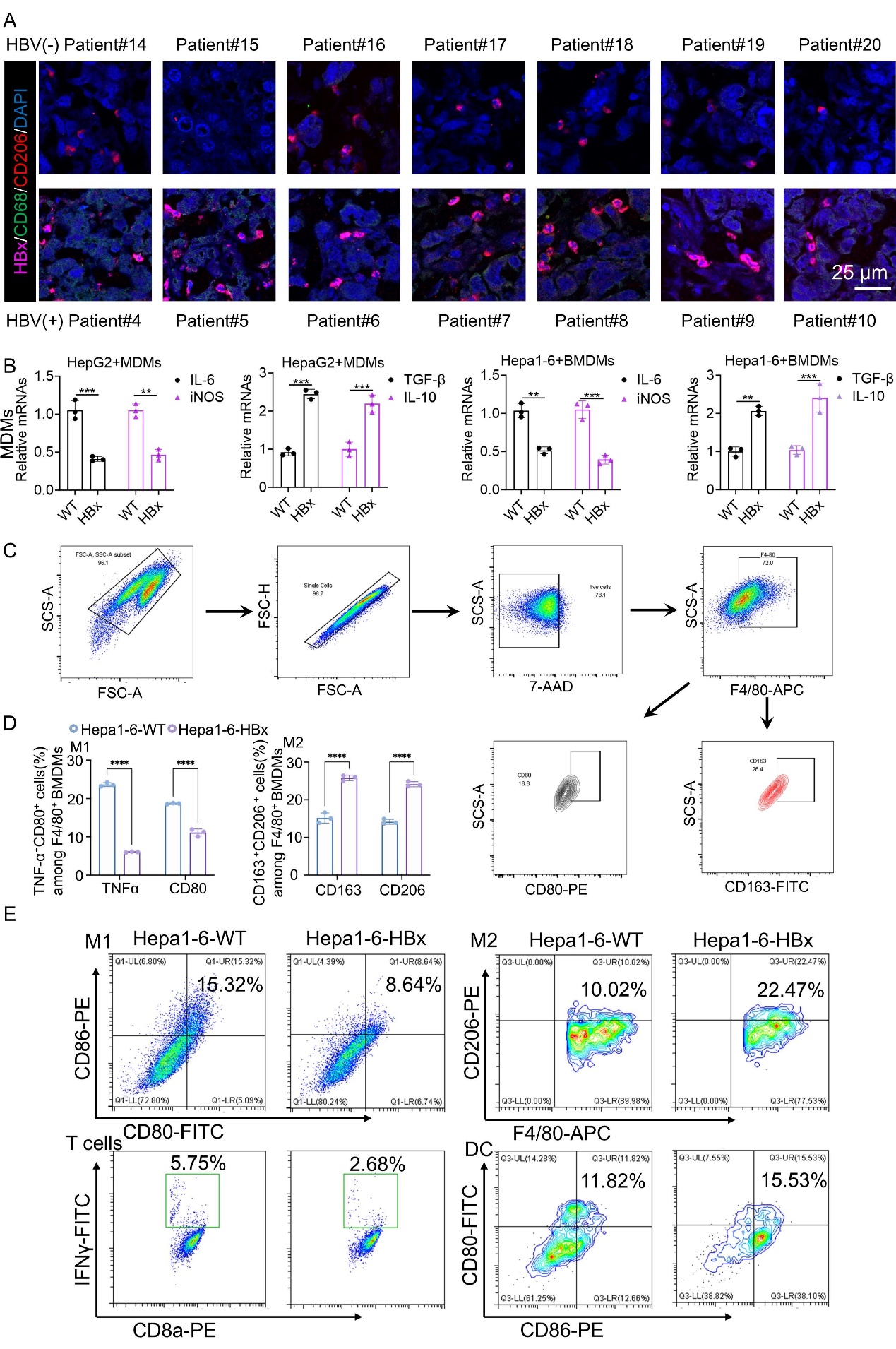


Figure S1 HBx-related tumor cells inhibit activation of the immune cell *in vitro*. (A) Representative images of CD68 and CD206 staining in patient-derived tissue samples from HBV (-) or HBV (+) HCC patients. HBx, purple; CD68, green; CD206, red; and nuclear staining (DAPI), blue. Scale bar: 25 μm. (B) RT-qPCR detection of IL-6, iNOS, TGF-β or IL-10 in BMDMs or MDMs treated with supernatants (Sup) of WT or HBx-positive cells (n=3). (C) Gating strategies for for analysis of the BMDMs. (D) Change of percentages of CD80^+^ or CD206^+^ among F4/80^+^ BMDM cells co-cultured with Hepa1-6-WT cells or Hepa1-6-HBx cells (n=3). (E) Percentages of splenetic CD80^+^CD86^+^ cells among F4/80^+^ M1 macrophages, CD206^+^ cells among F4/80^+^ M2 macrophages, IFNγ^+^ cells among CD8^+^ T cells, and CD80^+^CD86^+^ cells among CD11c^+^ DCs. Statistical analysis was carried out using two-way-ANOVA. **p < 0.01, ***p < 0.001, ****p < 0.0001. Error bars represent mean ± SD.


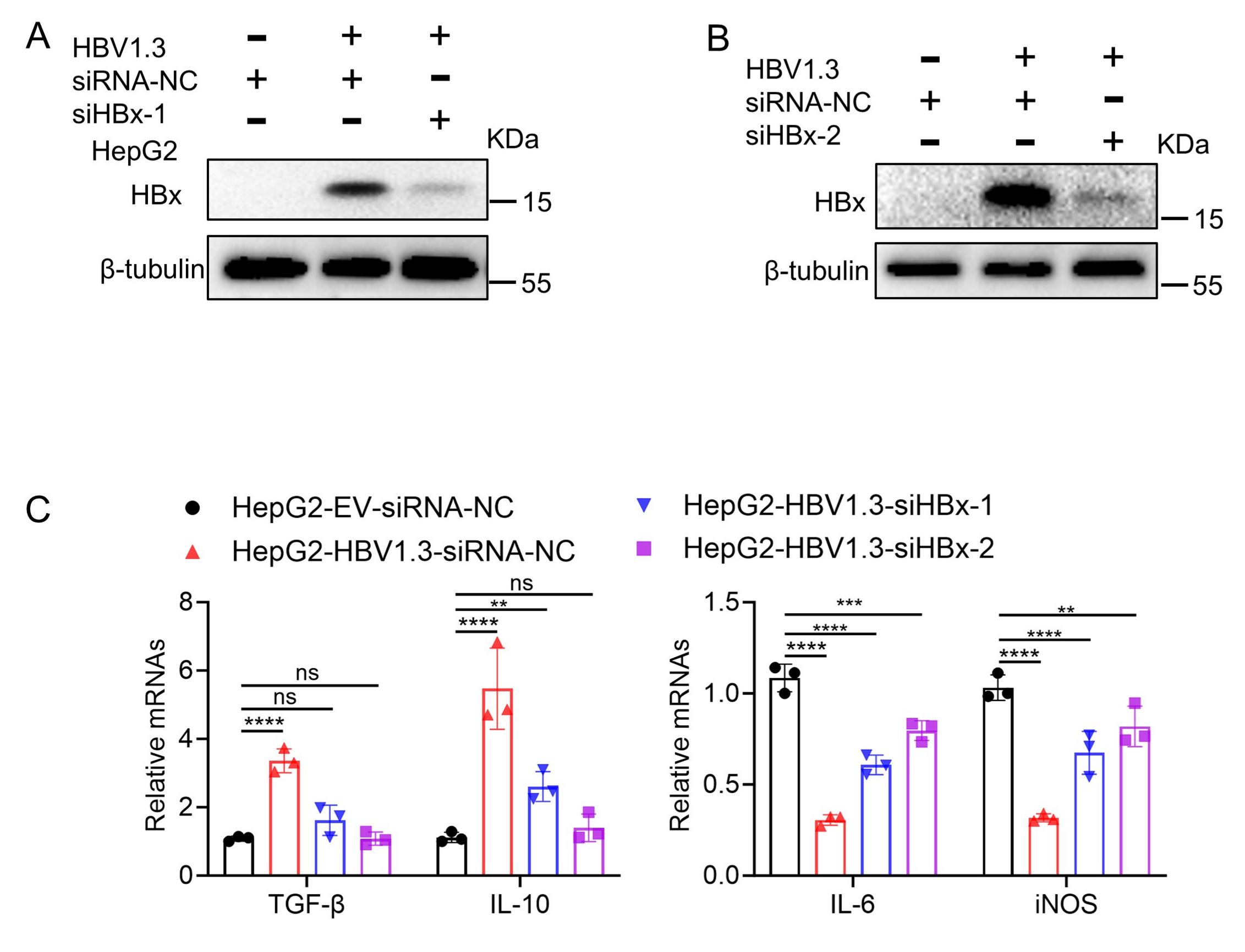


Figure S2 HBx is responsible for M2 macrophage polarization. (A and B) WB detection of HBx and β-tubulin in HBV 1.3-transfected HepG2 with *HBx* knockdown or not. (C) RT-qPCR evaluation of TGF-β, IL-10, IL-6, or iNOS mRNAs in MDMs treated with the Sup from HBV1.3-transfected HepG2 with *HBx* knockdown or not. Statistical analysis was carried out using two-way-ANOVA. ns, no significance, **p < 0.01, ***p < 0.001, ****p < 0.0001. Error bars represent mean ± SD.


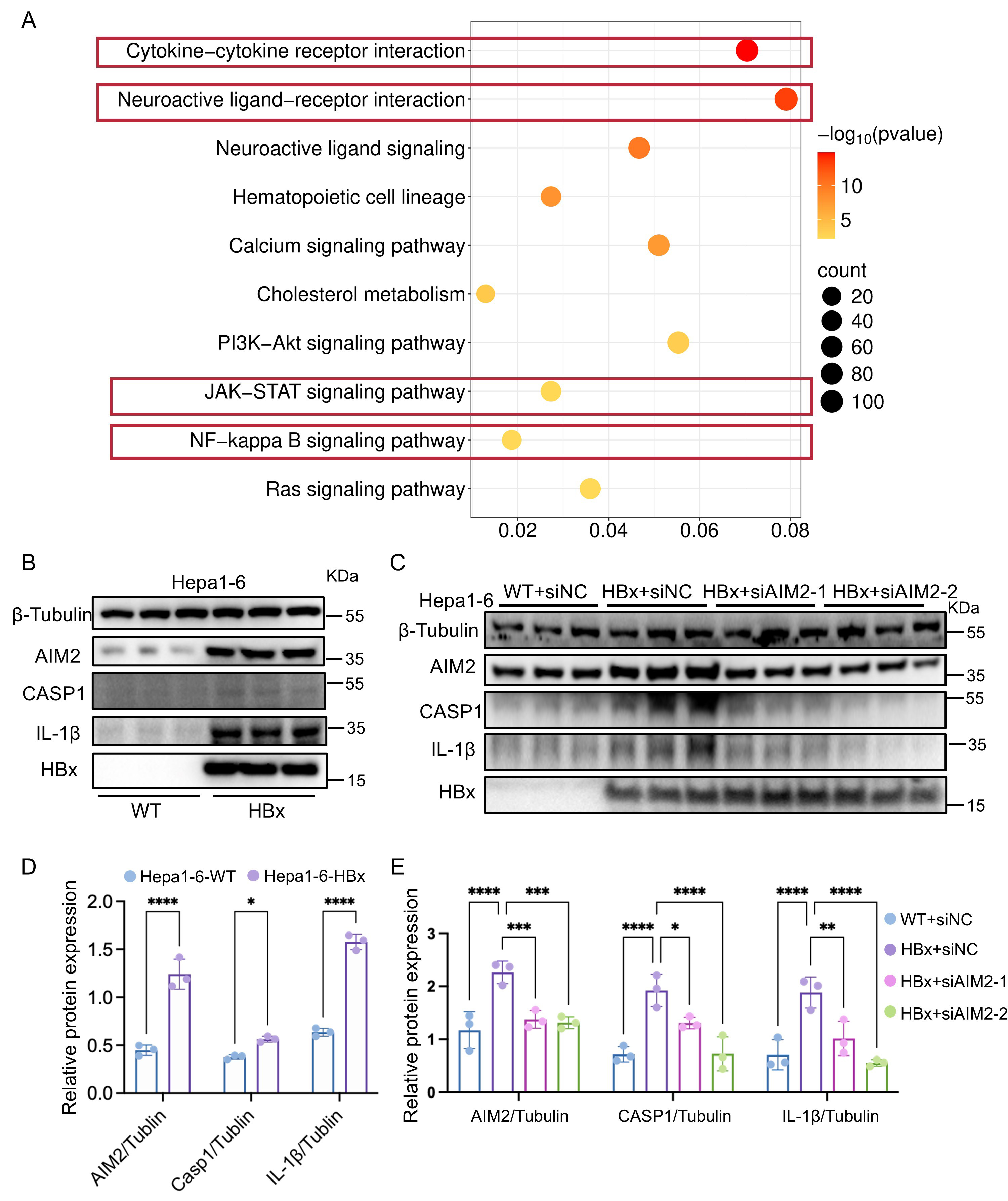


Figure S3 HBx induces activation AIM2 axis in tumor cells. (A) Significantly enriched KEGG pathways through KEGG pathway enrichment analysis of DEGs. Each bubble indicates an enriched KEGG term, where the red block diagram represents the interested KEGG pathway. (B-E) WB detection of AIM2, IL-1β, CASP1, HBx and GAPDH in WT and HBx-expressing Hepa1-6 with *AIM2* knockdown or not (B and C) Protein expression of AIM2, IL-1β, or CASP1 relative to GAPDH (n=3) (D and E). Statistical analysis was carried out using two-way-ANOVA. *p < 0.05, **p < 0.01, ***p < 0.001, ****p < 0.0001. Error bars represent mean ± SD.


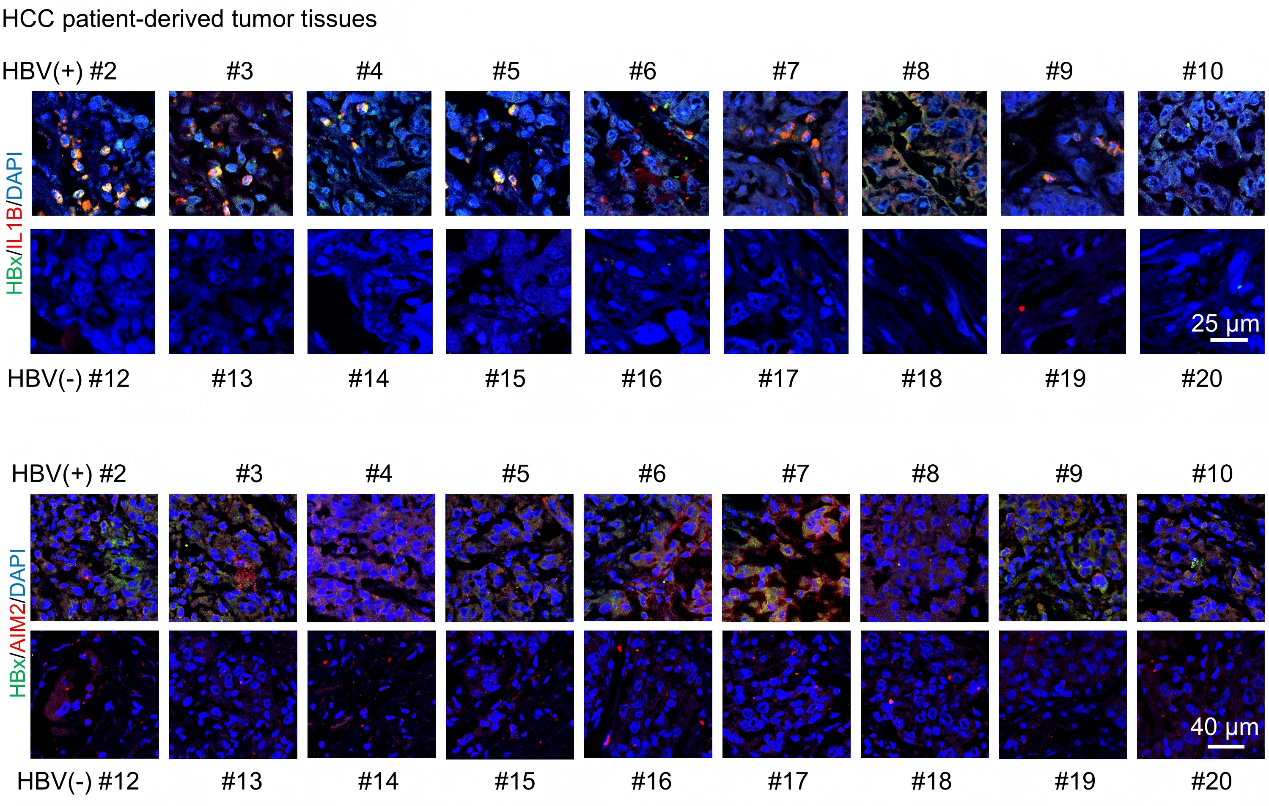


Figure S4 Representative images of IL-1β (red) or AIM2 (red) staining in patient-derived tissue samples from HBV (-) or HBV (+) HCC patients. Scale bar: 25 μm or 40 μm.


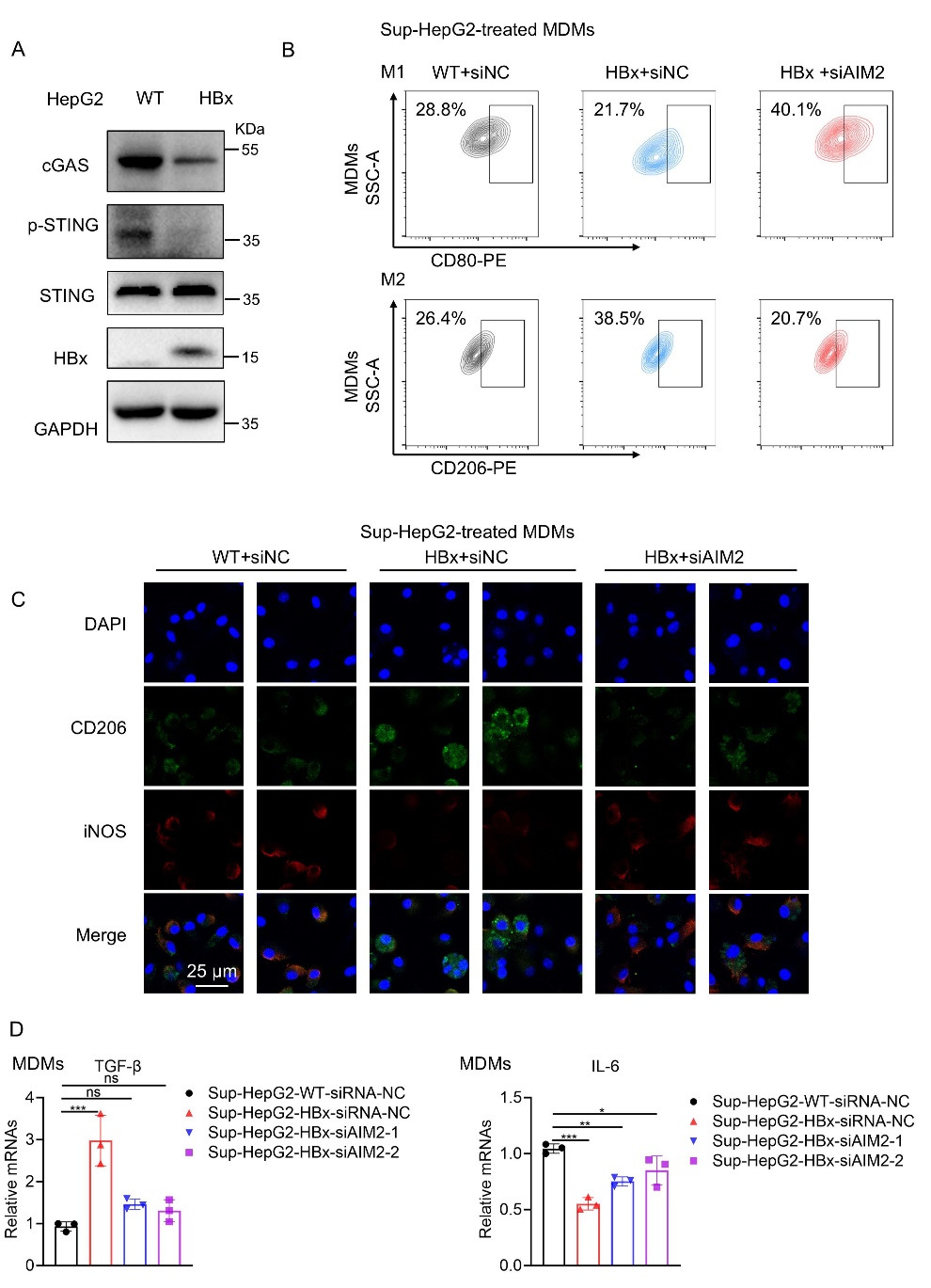


Figure S5 Tumor cell intrinsic HBx-AIM2 activation promotes macrophage M2 polarization. (A) WB detection of cGAS, p-STING, STING and GAPDH expression in HepG2-WT or -HBx cells. (B) Frequency of M1 type MDMs (CD80^+^ cells) or M2 type MDMs (CD206^+^ cells) after being treated with the Sup from control or *AIM2*-knockdown HepG2-HBx cells. (C) Representative images of CD206 and iNOS staining in MDMs treated with the Sup from control or *AIM2* knockdown HepG2 cells. CD206, green; iNOS, red; and DAPI staining for nucleus. The scale bar represents 25 μm. (D) RT-qPCR detection of TGF-β or IL-6 in MDMs treated with the Sup from control or *AIM2* knockdown HepG2 cells. Statistical analysis was carried out using one-way-ANOVA. ns, no significance, *p < 0.05, **p < 0.01, ***p < 0.001. Error bars represent mean ±SD.


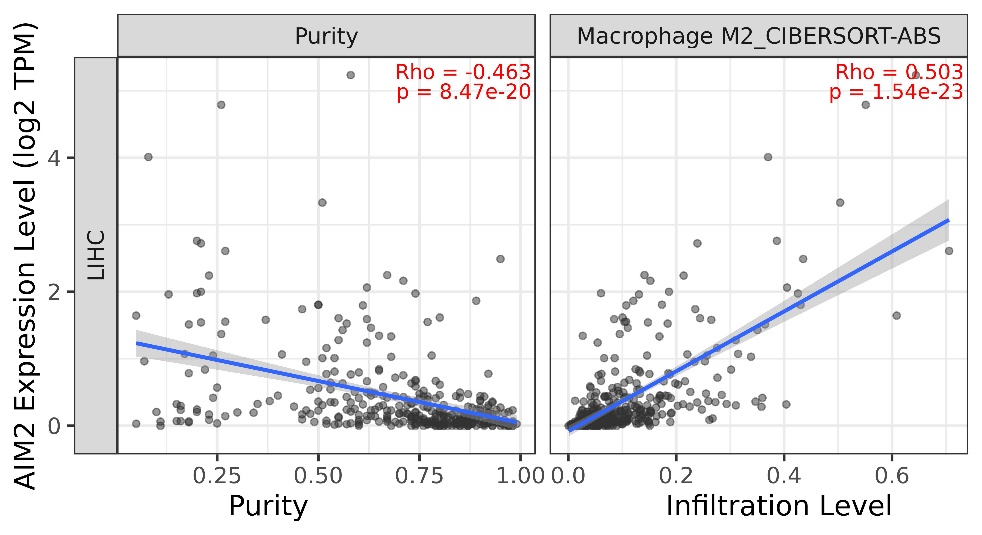


Figure S6 Correlation analysis between AIM2 expression and the level of M2 tumor-associated macrophage infiltration in liver cancers, generated using the TIMER2.0 algorithm.


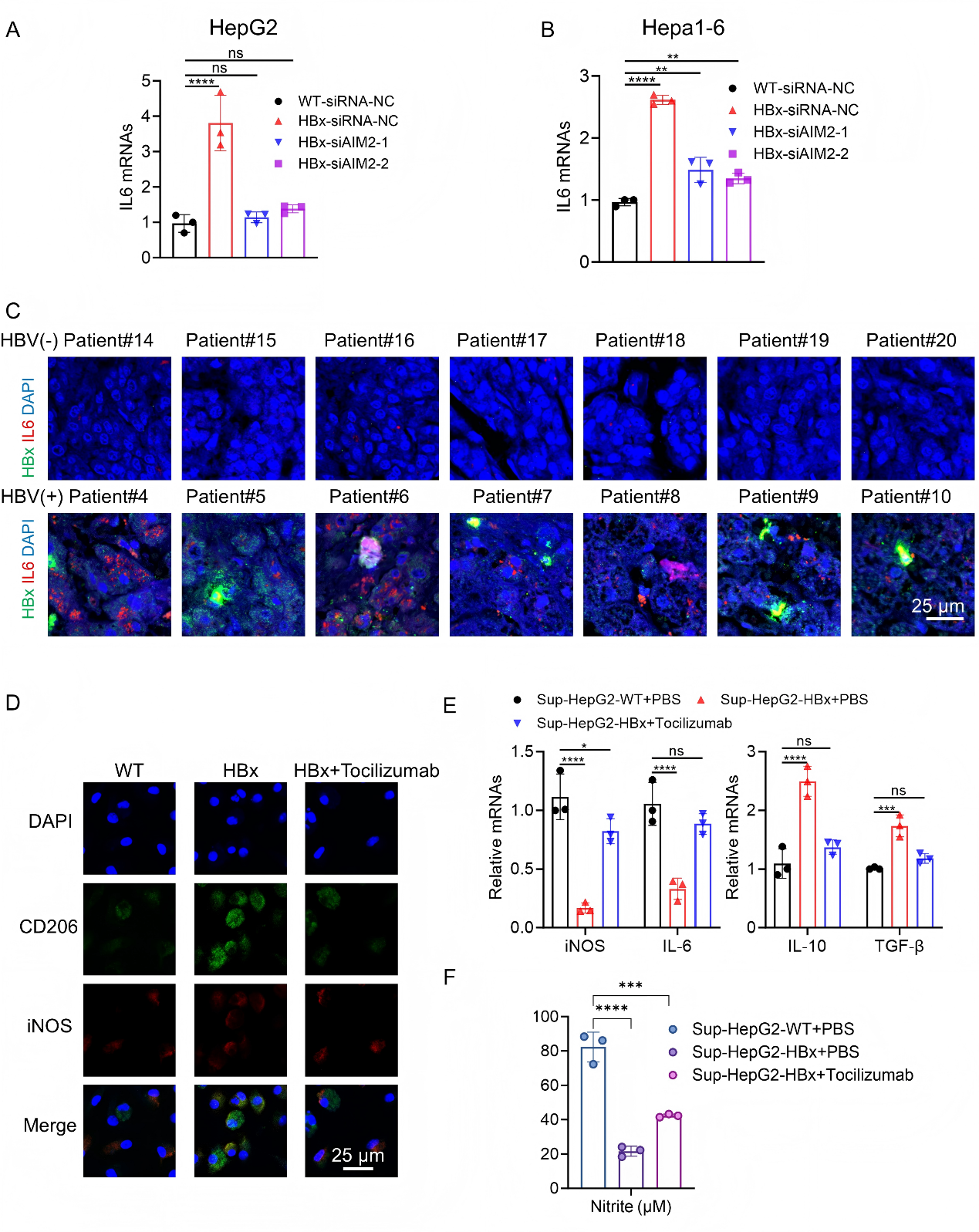


Figure S7 HBx triggers AIM2-IL6 activation responsible for M2 polarization induction. (A and B) RT-qPCR detection of IL-6 mRNAs in control or *AIM2* knockdown cells. (C) Representative images of IL-6 and HBx staining in the clinical samples from HBV (-) or HBV (+) HCC patients. HBx, green; IL-6, red; and DAPI staining for nucleus, blue. The scale bar represents 25 μm. (D) Representative images of CD206 or iNOS staining in MDMs treated with the Sup from HepG2-WT or HepG2-HBx cells in the presence or absence of Tocilizumab. CD206, green; iNOS, red; and DAPI staining for nucleus. The scale bar represents 25 μm. (E) RT-qPCR detection of TGF-β, IL-10, IL-6, or iNOS in MDMs treated with the Sup from the indicated tumor cells in the presence of Tocilizumab or not. (F) Nitrite measured in media from MDM culture by Griess assay following specific treatments and subsequent LPS/IFNγ stimulation. Statistical analysis was carried out using one-way-ANOVA or two-way-ANOVA. ns, no significance, *p < 0.05, **p < 0.01, ***p < 0.001, ****p < 0.0001. Error bars represent mean ± SD.


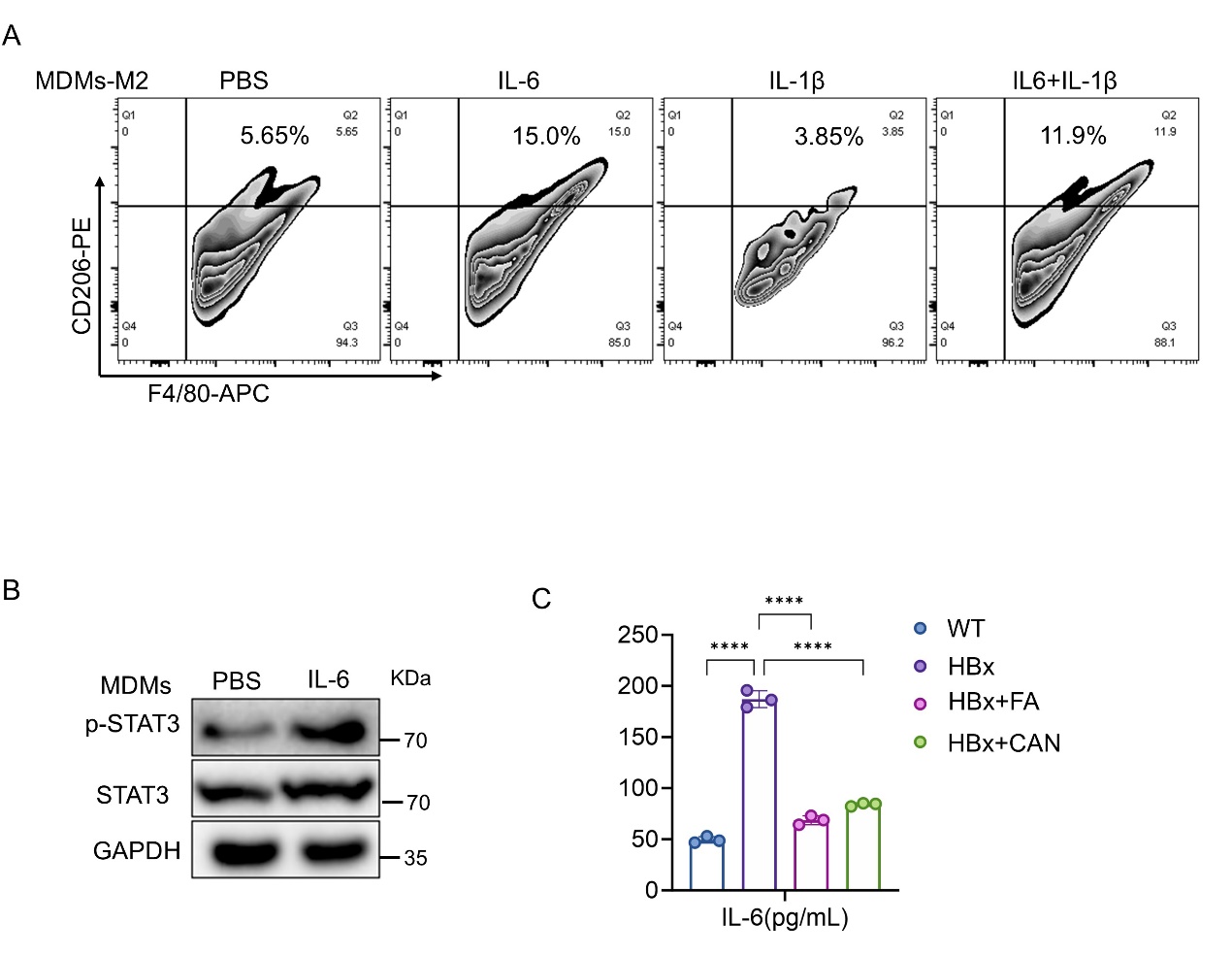


Figure S8 IL6 induces M2 polarization. (A) Percentages of CD80^+^ F4/80^+^ M1 macrophages, and CD206^+^ F4/80^+^ M2 macrophages pretreated with PBS, IL-6, IL-1β or IL6 plus IL-1β. (B) WB examination of abundance of p-STAT3, STAT3, and GAPDH in MDMs treated with IL-6 or not. (C) ELISA detection of IL-6 from the Sup of HepG2-WT and HepG2-HBx cells in the presence of FA, CAN or not. Statistical analysis was carried out using one-way-ANOVA. ****p < 0.0001. Error bars represent mean ± SD.


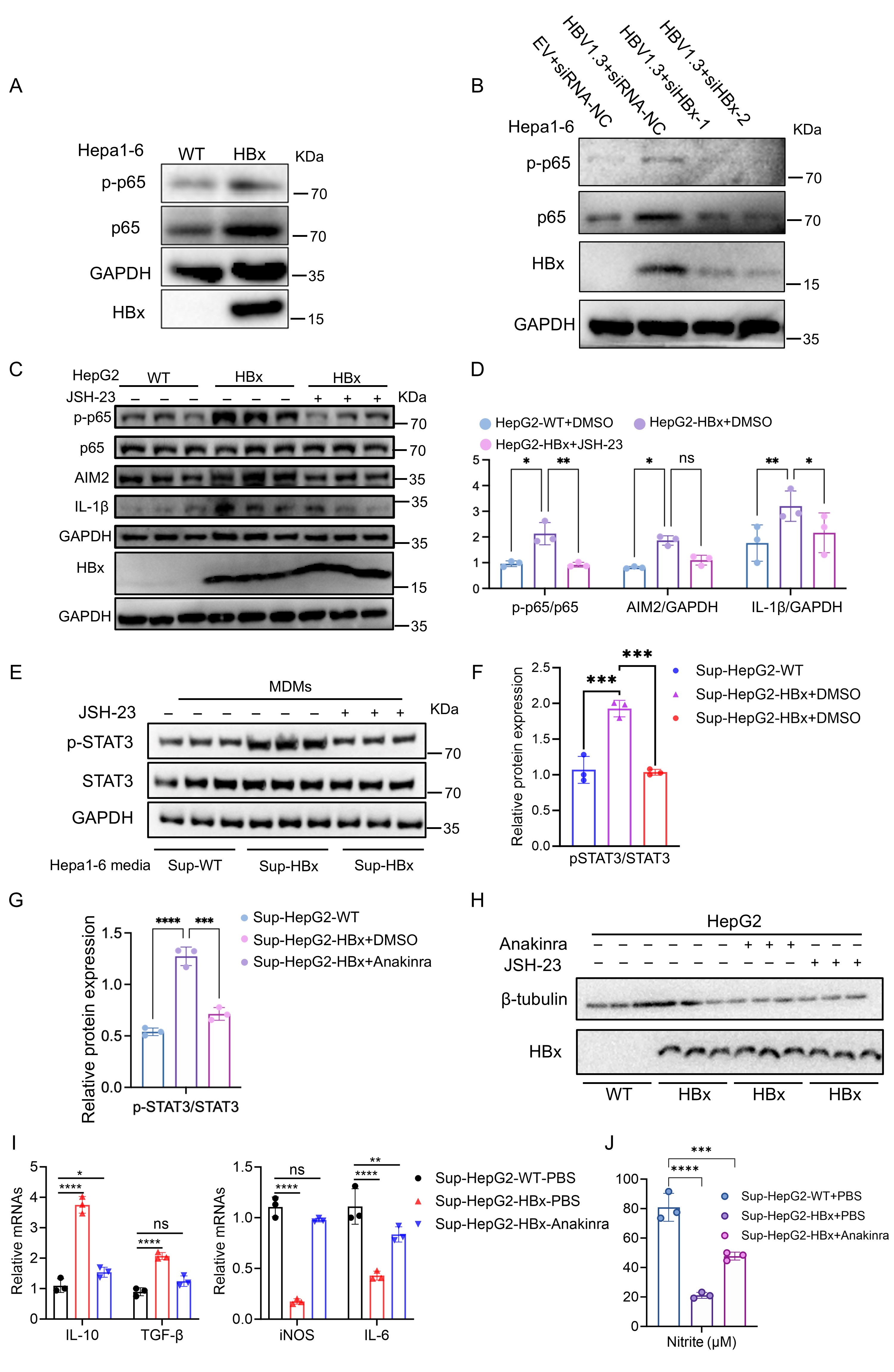


Figure S9 HBx-induced p65 activation elicits M2 macrophage polerization. (A) WB examination of p65 phosphorylation in HepG2-WT and HepG2-HBx cells. (B) WB analysis of p-p65, p65, HBx or GAPDH in HBV 1.3 plasmid-transfected Hepa1-6 cells with *HBx* knockdown or not. (C and D) WB detection of p-p65, AIM2, IL-β, HBx or GAPDH expression in WT or HBX-related Hepa1-6 cells treated with DMSO or JSH-23 (C). Protein expression of p-p65 relative to p65, AIM2 and IL-β relative to GAPDH in those treated cells (D). (E and F) WB examination of abundance of p-STAT3, STAT3 or GAPDH in MDMs treated with the Sup from HepG2-WT or HepG2-HBx in the presence of JSH-23 or not (E). Protein expression of p-STAT3 relative to STAT3 in those treated cells (F). (G) Protein expression of p-STAT3 relative to STAT3 in HepG2 cells with the indicated treatment. (H) WB examination of HBx or GAPDH expression in WT or HBX-related HepG2 cells treated with DMSO, JSH-23 or Anakinra. (I) RT-qPCR evaluation of TGF-β, IL-10, IL-6, or iNOS mRNAs in MDMs treated with the Sup from the indicated tumor cells in the presence of Anakinra or not. (J) Nitrite measured in media from MDM culture by Griess assay following specific treatments and subsequent LPS/IFNγ stimulation. Statistical analysis was carried out using one-way-ANOVA, or two-way-ANOVA. * ns, no significance, *p < 0.05, **p < 0.01, ***p < 0.001, ****p < 0.0001. Error bars represent mean ± SD.


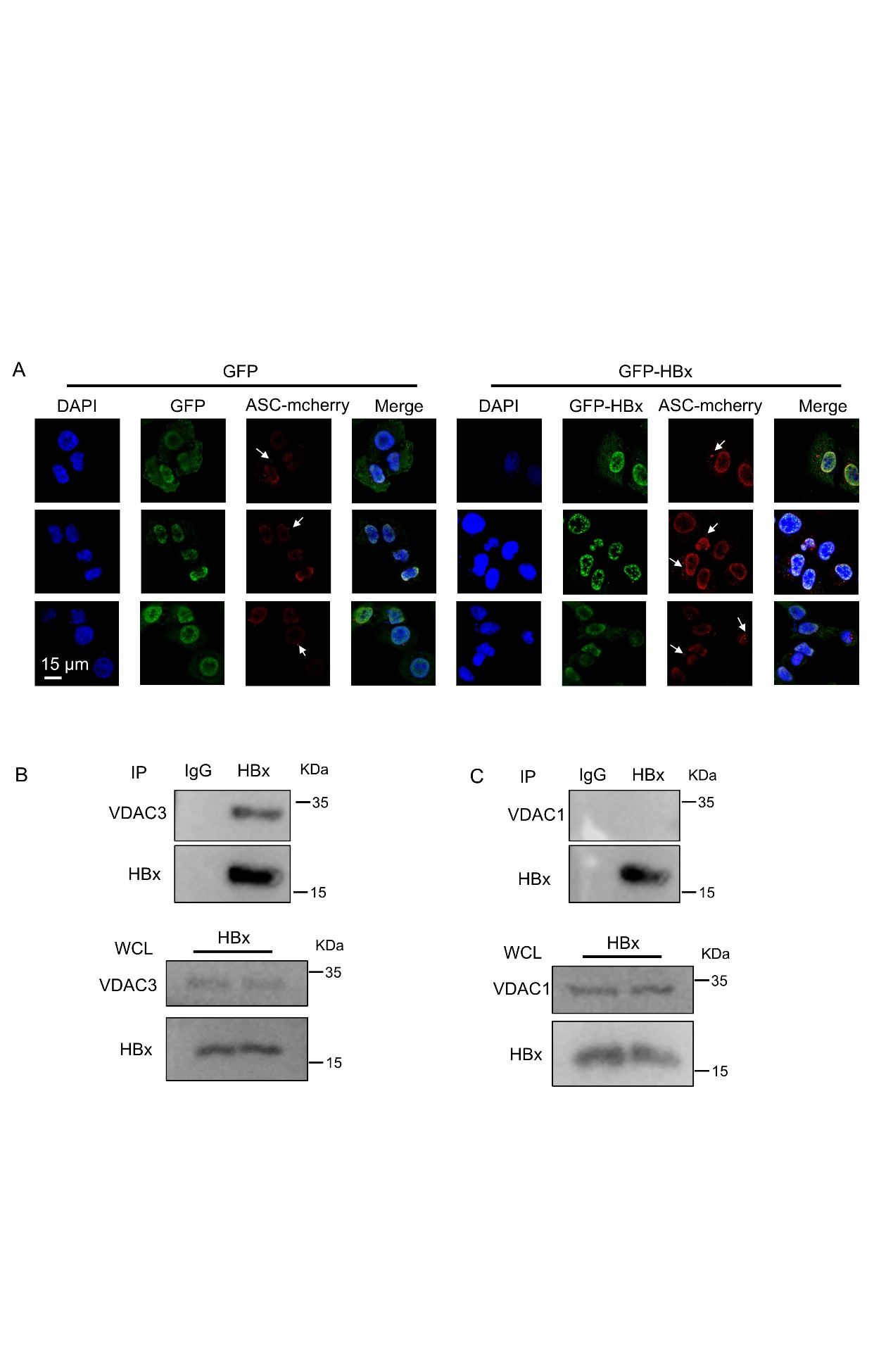


Figure S10 (A)Representative images of ASC staining in HepG2 cells transfected with GFP-EV or GFP-HBx-expressing plasmid. GFP, green; ASC, red; and DAPI staining for nucleus. The scale bar represents 25 μm. MFI of ASC speck formation in those treated HepG2 cells. Statistical analysis was carried out using Student’s t test, **p < 0.01. Error bars represent mean ± SD. (B and C) WB detection of HBx, VDAC3 or VDAC1 expression in WCLs or the protein immunoprecipitants using anti-HBx antibody from HBx-Flag plasmid-transfected HepG2 cells.


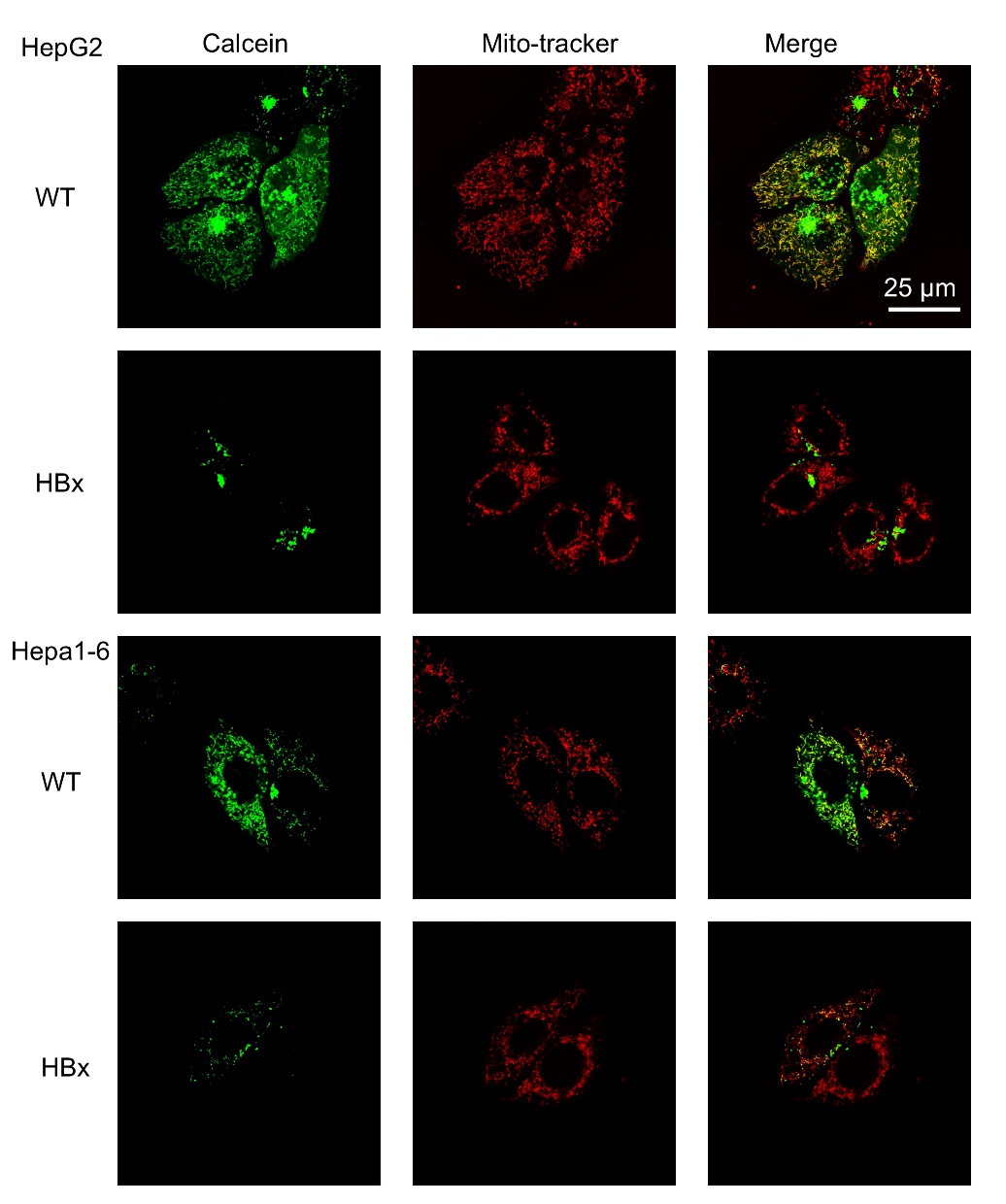


Figure S11 HBx causes the mPTP opening. Representative images of Calcein and mitochondria staining in WT or HBx-related tumor cells. Calcein, green and Mito-tracker, red. The scale bar represents 25 μm.


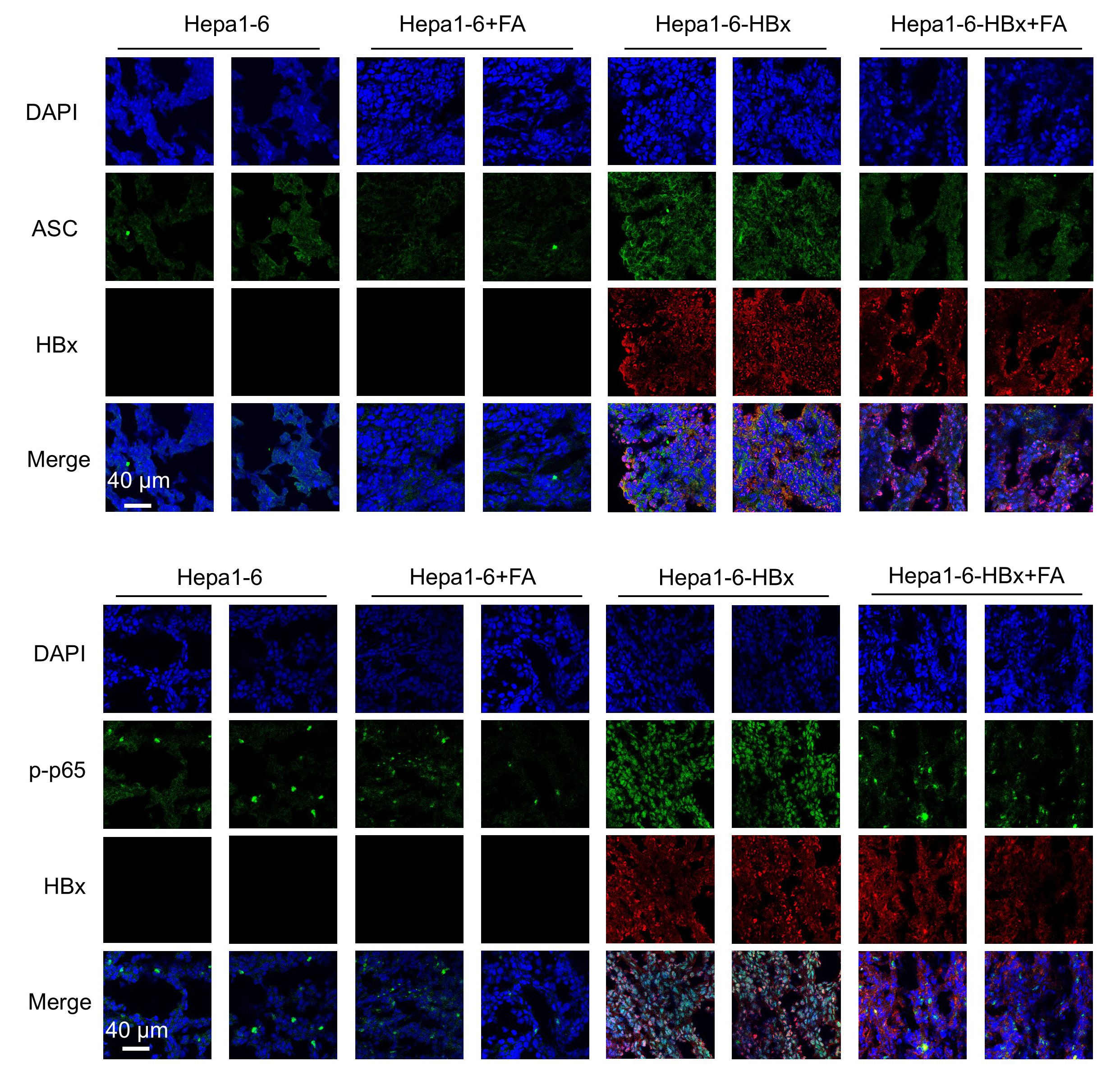


Figure S12 Representative images of HBx (red), ASC (green) or p-p65 (green) staining in WT- or HBx-related Hepa1-6 tumors in the presence of FA or not. The scale bar represents 40 μm.


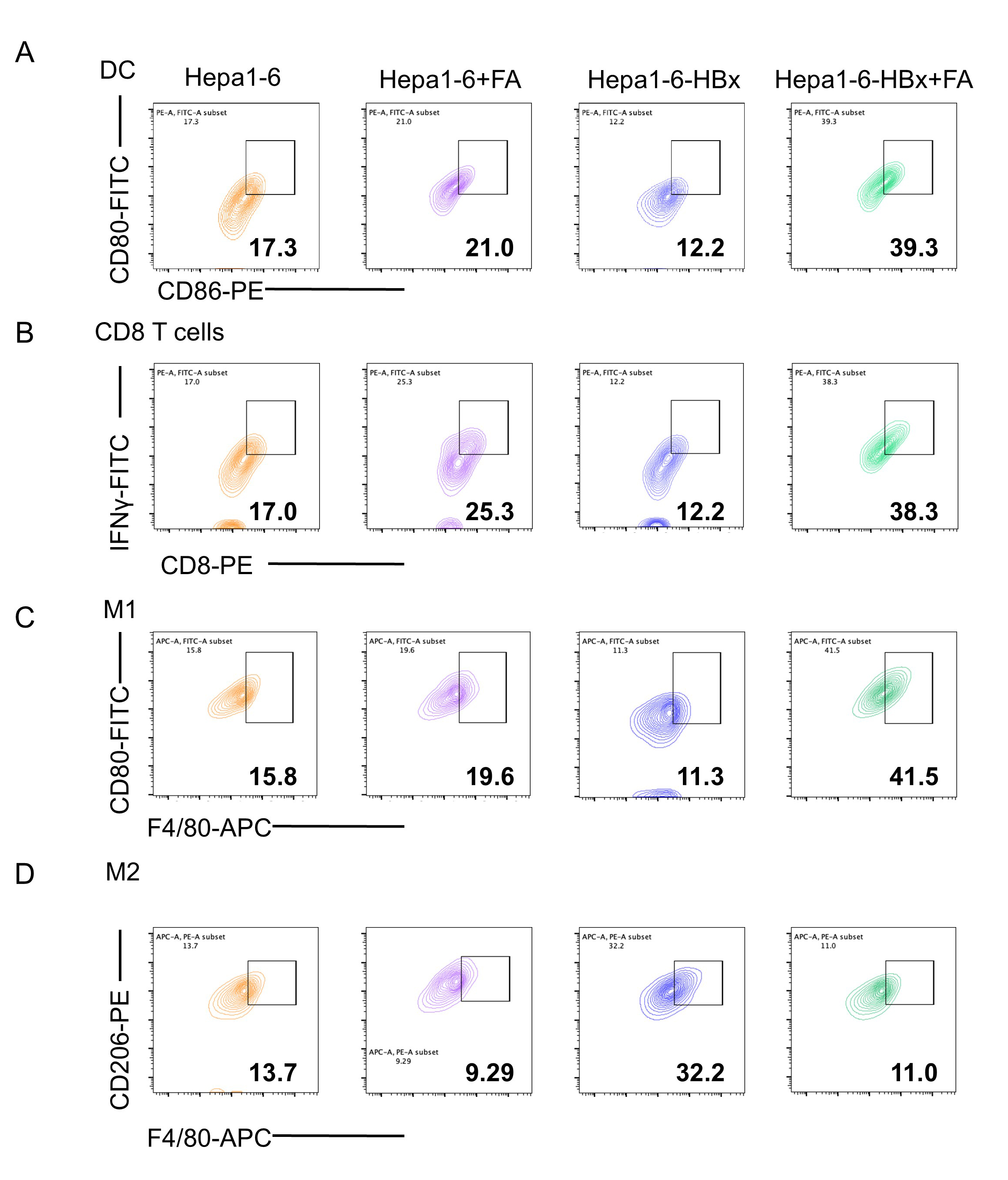


Figure S13 Frequency of intratumoral matured DC, cytotoxic CD8 T cells, M1 macrophages or M2 macrophages from murine tumors receiving different treatments.


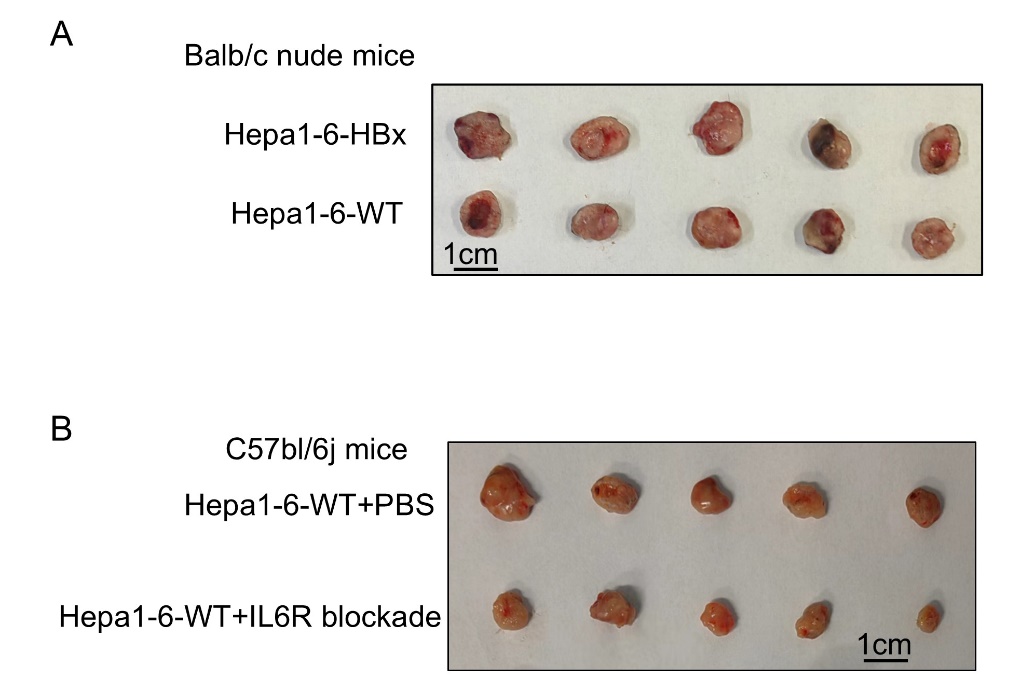


Figure S14 HBx promotes murine tumor development dependent on antitumor immunity. (A) Changes of tumor size from Hepa1-6-HBx and Hepa1-6-WT tumor-bearing immunocompromised Balb/c nude mice models. (B) Changes of tumor size from Hepa1-6 WT tumor bearing C57bl/6j mice models treated with PBS or IL6 blockade. The scale bar represents 1 cm.


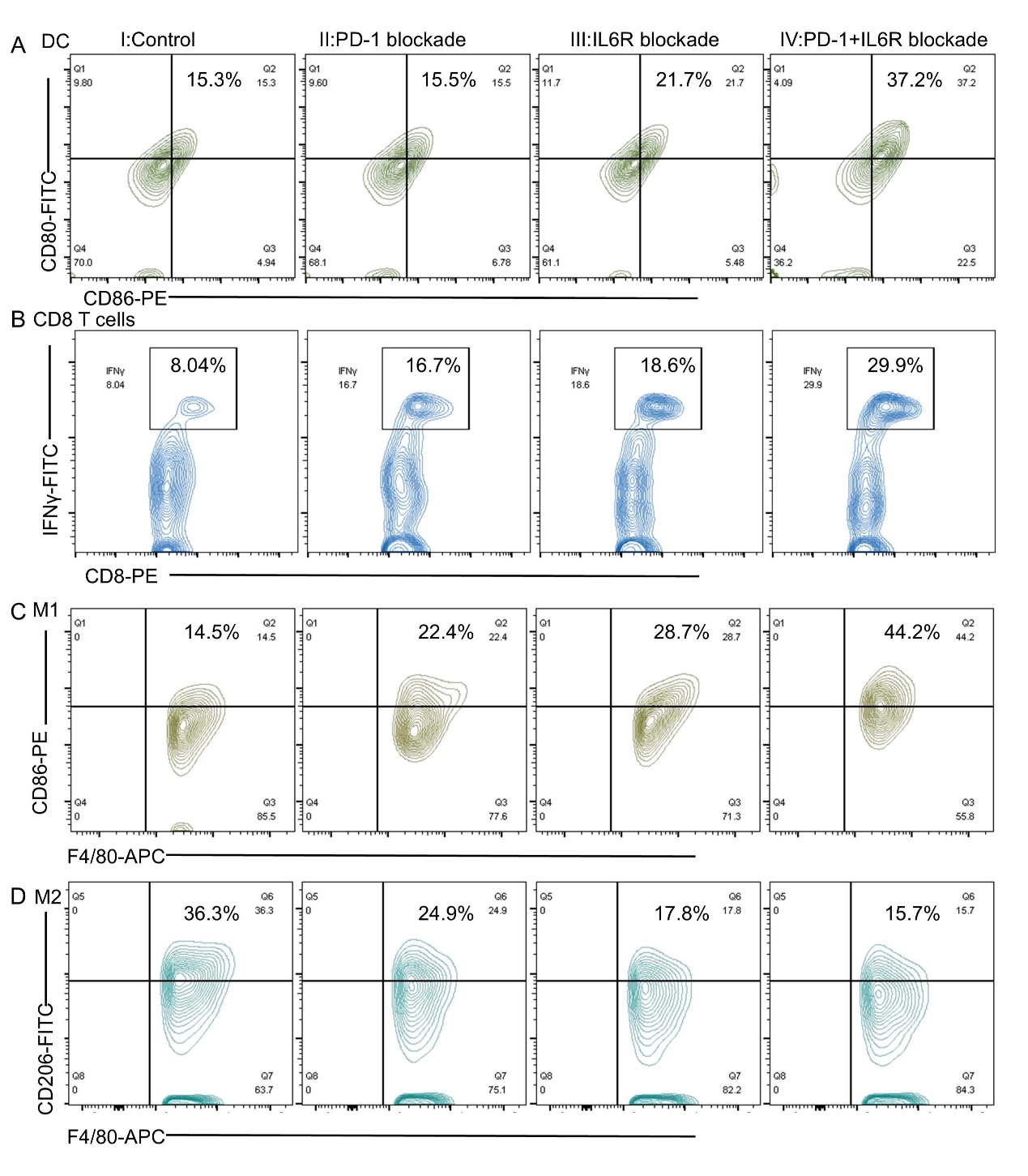


Figure S15 (A-D) FCA analysis of DC maturation (A), M1 TAMs (B), M2 TAMs (C), and CD8^+^ T cell activation (D) from Hepa1-6-tumor bearing mice with different treatments.
